# A dynamical model for the basal ganglia-thalamo-cortical oscillatory activity and its implications in Parkinson’s disease

**DOI:** 10.1007/s11571-020-09653-y

**Published:** 2020-11-25

**Authors:** Eva M. Navarro-López, Utku Çelikok, Neslihan S. Şengör

**Affiliations:** 1 School of Mathematics and Computer Science, Faculty of Science and Engineering, University of Wolverhampton, Alan Turing Building, Wulfruna Street, Wolverhampton, WV1 1LY UK; 2 Biomedical Engineering Department, Boğaziçi University, 34342 Beşiktaş, Istanbul Turkey; 3 Department of Electronics and Telecommunication Engineering, Istanbul Technical University, 34469 Maslak, Sariyer, Istanbul Turkey

**Keywords:** Basal ganglia, Brain oscillations, Computational and mathematical models, Parkinson’s disease, Spiking neural networks, Neuroplasticity, Adaptive dynamical evolution, Self-organisation, Collective behaviour

## Abstract

We propose to investigate brain electrophysiological alterations associated with Parkinson’s disease through a novel adaptive dynamical model of the network of the basal ganglia, the cortex and the thalamus. The model uniquely unifies the influence of dopamine in the regulation of the activity of all basal ganglia nuclei, the self-organised neuronal interdependent activity of basal ganglia-thalamo-cortical circuits and the generation of subcortical background oscillations. Variations in the amount of dopamine produced in the neurons of the substantia nigra pars compacta are key both in the onset of Parkinson’s disease and in the basal ganglia action selection. We model these dopamine-induced relationships, and Parkinsonian states are interpreted as spontaneous emergent behaviours associated with different rhythms of oscillatory activity patterns of the basal ganglia-thalamo-cortical network. These results are significant because: (1) the neural populations are built upon single-neuron models that have been robustly designed to have eletrophysiologically-realistic responses, and (2) our model distinctively links changes in the oscillatory activity in subcortical structures, dopamine levels in the basal ganglia and pathological synchronisation neuronal patterns compatible with Parkinsonian states, this still remains an open problem and is crucial to better understand the progression of the disease.

**Electronic supplementary material:**

The online version of this article (10.1007/s11571-020-09653-y) contains supplementary material, which is available to authorized users.

## Introduction

The basal ganglia (BG) are subcortical structures located in the human forebrain and are buried deep into the brain. Experimental studies have revealed that the BG exert an excitatory influence on a number of cortical areas by driving glutamatergic projections from the thalamus. Neurophysiological and anatomical studies have also shown that inputs coming from different regions of the cortex are directed to the thalamus through the BG circuits and, finally, projected back to the cortex from which the circuit is originated. Topographically-organised projections of the cortical-BG-thalamic loops mediate specific functions for task-related stimuli (Aron 2007; Surmeier 2013). Moreover, projections of the cortical-BG-thalamic loops have been suggested to act as a gating mechanism in action selection, as well as for information selection in planning, cognition, and reinforcement learning (Frank and Badre 2011; van Schouwenburg et al. 2013).

During a cognitive or motor task, cortical areas are engaged with the dorsal and rostral caudate nucleus, which is densely innervated by dopaminergic neurons, most of which originate from the substantia nigra pars compacta (SNc) and the ventral tegmental area (Joksimovic et al. 2009). The principal neurons within the striatum—that is, medium spiny neurons (MSNs)—receive glutamatergic inputs from cortical regions and dopaminergic inputs from the SNc, and convey the primary inputs to the BG system. MSNs are divided into two major subtypes depending on their axonal targets and dopamine (DA) receptors, mainly $D_1$ and $D_2$ receptors (Le Moine et al. 1991). The distinction between the response of MSNs to the neurotransmitter DA gives rise to two segregated pathways: the direct and the indirect pathways. The balance between these pathways is regulated by the level of DA. An abundance of DA ‘releases the brakes’ over the dorsal thalamus, resulting in neuronal activation. An increased activity within the thalamus ‘opens the gates’ for information relay and allows cortical representation of the cue. Therefore, the BG can be interpreted as a ‘switching control mechanism’ which is modulated by the DA (Gerfen et al. 1995). The direct and indirect pathways of the BG must work together, allowing appropriate action selection computations to take place via parallel networks, and regulating the activity within the thalamus. The thalamus works as a translator of the message received from the BG rather than just a relay element. Dysfunctions within the BG network are shown to be related to deficits in the execution of specific cognitive and motor tasks, giving rise to abnormal patterns compatible with neurological disorders such as Parkinson’s disease, Huntington’s disease and schizophrenia (Gerfen 2000; Keshavan et al. 2003).

This paper focuses on Parkinson’s disease (PD) and its link to BG activity. One of the manifestations of PD is a deficiency in dopamine production in the neurons of the SNc (Obeso et al. 2004, 2008). It develops gradually and mainly affects motor functions, including: resting tremor, akinesia, bradykinesia and rigidity, plus dyskinesia after dopamine-related treatment (Guridi et al. 2012). Additionally, it affects cognitive abilities, like memory and attention. Deficits caused by PD are challenging to observe in laboratory experiments. Neuro-computational models can help improve the analysis of experimental results and reveal new characteristics about the evolution of the disease and their physical and cognitive consequences.

One well-established functional theory of PD refers to the changes in the oscillatory activity patterns, altered power spectra, and pathological synchronisation of neurons in the BG, which are observed in animal models of PD and human patients with PD (Boroud et al. 2005; Hutchison et al. 2004; Obeso et al. 2004, 2008). However, the relationship between these changes and the severity of PD has not been adequately explored. Electrophysiological recordings in PD patients have demonstrated that different nuclei of the BG fire with an exaggerated synchronisation at a specific frequency range of neuronal activity; particularly, at beta frequency-band (13–30 Hz) (Boroud et al. 2005; Brown et al. 2001). In PD, the BG, along with the thalamus, have a tendency to resonate at a certain frequency interval in which pathological symptoms are intensified (Brazhnik et al. 2016). To generate an appropriate selection mechanism, each BG nucleus should oscillate in a particular frequency range, besides enduring a certain level of synchrony. Inadequacy of dopamine drive in the BG, particularly in the subthalamic nucleus and the globus pallidus, leads to impaired activity at multiple levels within this circuit that eventually results in pathophysiological processes underlying PD. These data suggest that the strength of locally and spatially-distributed oscillatory activity, as well as the strength of these neural oscillations, may provide valuable insights into the pathophysiology of Parkinsonian states and offer a more accurate surgical targeting or deep brain stimulation (DBS) parameter selection for PD treatment.

Mathematical and computational models of the BG-dopamine system have provided accurate predictions on BG function (Frank 2005; Gurney et al. 2001; Mandali et al. 2015; Prescott et al. 2006; Sengör and Karabacak 2015), PD-related alterations in the network (McCarthy et al. 2011; Muralidharan et al. 2014), and DBS treatment (Neumann et al. 2018; Rouhollahi et al. 2019; Schiff 2012), as well as novel theories for PD (Terman et al. 2002). A detailed survey and discussion on DBS can be found in Little and Bestmann (2015). Furthermore, computational models of the BG can provide information on levodopa medication (Baston et al. 2016) and on the role of dopamine in Parkinsonian akinesia and tremor to explore new therapies for PD (Caligiore et al. 2019). Some of these mathematical models make use of detailed neuron models (McCarthy et al. 2011; Terman et al. 2002), while some others make use of simple models like the leaky-integrate-and-fire model with a sigmoid-like function to reproduce the mean firing rate of each neural substructure in the BG network (Baston et al. 2016; Frank 2005; Gurney et al. 2001; Neumann et al. 2018; Prescott et al. 2006; Rouhollahi et al. 2019; Sengör and Karabacak 2015). Other models predict BG behaviour by using point-neuron models, such as integrate-and-fire and Izhikevich’s models. However, instead of considering the approximation of the mean firing rate with a sigmoid function, they use a substantial number of neurons to model each neural structure (Caligiore et al. 2019; Mandali et al. 2015).

Finally, the neuron models proposed in Humphries et al. (2009) are able to reproduce the effect of DA on striatal neurons with $D_1$ and $D_2$ receptors. During action selection, the model enhances the difference between the cortical inputs to allow the BG to select between competing information. The model in Thibeault and Srinivasa (2013) uses a simple neuron model to simulate the BG action selection mechanism. It investigates the impairments in BG selection capabilities caused by PD and explains how they are restored by DBS in terms of firing rates, firing patterns, and synchronisation characteristics. This study provides a power-efficient computational model suitable for large-scale network simulations that might be used to further improve the efficacy of DBS therapy. The model presented in Frank et al. (2001) considers the BG circuitry as a mechanism that enables cortical functions to take place at appropriate times. In other words, the BG decide ‘when to do’ and the cortex knows ‘what to do’.

Inspired by the findings in Humphries et al. (2009), Thibeault and Srinivasa (2013), Frank et al. (2001), we propose a dynamical model for the BG network in order to investigate dynamical aspects of the BG under normal DA conditions, and in scenarios compatible with Parkinsonian states. Our model takes DA into consideration as a modifiable control parameter. This is pertinent since PD is associated with a decreased amount of DA, below the normal level. Dopamine degradation in PD occurs at two modalities: tonic and phasic. The tonic DA release regulates the steady-state extracellular DA concentration, whilst the phasic DA release represents the rapid and transient DA response when an external stimulus is applied in the cortex (Schultz 2016). In this sense, we consider both slow and fast components of DA release in the network model as tonic and phasic DA levels, respectively. Defining how the DA levels in the BG circuit are related to hypo-activity and hyper-synchrony in the spatially-distributed brain regions is critical for understanding PD-related processes.

The advantages of the proposed model are twofold. First, the model is able to reproduce a broad electrophysiological repertoire of spiking patterns despite its simplicity. The diversity of the spiking characteristics is achieved by using an extended version of Izhikevich’s neuron model for single cells, whose parameters are determined by examining the dynamic response of the neuron models in the phase space and by carrying out a bifurcation analysis. This is important, because the spiking patterns of individual neurons are key to uncover the significance of the local field potential (LFP) oscillations generated by the BG network. Second, the network model consisting of the BG nuclei and the thalamus can reproduce experimentally-observed LFP oscillation features which are critical for the BG gating in scenarios built to illustrate healthy and PD-related behaviours. The proposed model confirms the onset of different dynamical collective behaviours of the BG produced by different levels of DA, which, at the same time, are associated with different frequency bands of the neural oscillations generated in the BG and the thalamus. The importance of the oscillatory activity patterns to control brain network states and the switching mechanisms in different phases has already been established (Navarro-López et al. 2016; Schmidt et al. 2013; Torres et al. 2015) for different brain processes. Our results represent a stepping stone to better understand the mechanisms behind the different frequency-band oscillations observed during BG functions. The main contribution of this work is to propose a modelling framework—simple enough even for large-scale neuromorphic hardware applications—which clarifies the role of the BG in the generation of subcortical oscillations associated with different DA levels.

The rest of the paper is organised as follows. “A dynamical model of the basal ganglia-thalamo-cortical network” section describes the models and simulations for the single neurons of the neural structures considered. Particularly, each nucleus of the BG, the posterior cortex and the thalamus. Additionally, we briefly explain how the BG-thalamo-cortical network model is built by interconnecting the single-neuron models. In order to clarify how the simulation results have been obtained and to ensure the reproducibility of our results, a companion paper as supplementary material is attached, with the equations and parameter values used for all the types of single-neuron models, and for the interconnections between neurons and populations of neurons. In “Network model results” section, we demonstrate how the simulation results of our mathematical-computational network model, under different conditions, can reproduce key selection capabilities: (1) in normal conditions where the DA is within a healthy range, and (2) in PD conditions where both phasic and tonic DA levels are present. Conclusions are given in the last section. The simulations presented in this paper have been done in MATLAB.

## A dynamical model of the basal ganglia-thalamo-cortical network

In this section, we describe a dynamical model of the BG-thalamo-cortical network that has been designed following empirically-validated data of the electrophysiology and anatomy of the network. Our simulation results are qualitatively compatible with the dynamical neuronal patterns and behaviours observed in real situations, which have been adequately referenced and explained throughout the paper, especially in the Introduction. With this, we ensure that the model is a reliable approximation of the system under study.

We begin with the BG, which is a collection of interconnected central grey nuclei with various cell types and diverse spiking characteristics. The dynamics of the BG nuclei are modulated by presynaptic afferents together with the neurotransmitter dopamine. Inside these nuclei, dopamine acts as a ‘gate holder’ for the cortical signals to pass through. Once the dopaminergic signal is received, a cascade of internal communication within the neural structures, which gives rise to oscillatory LFP activity at specific frequency bands, is required for the ‘gates to open’ appropriately. Four main structures of the BG nuclei are considered in the proposed model: the striatum, the external and internal segments of the globus pallidus (GPe, GPi), and the subthalamic nucleus (STN).

We then include a model for the posterior cortex, where the sensory information—represented as an external input—arrives and is passed as an input to the BG structures. Finally, a model for the thalamus is integrated into the network model. In the thalamus, the cortical signals are mapped onto after having been channeled through the BG loops.

**Fig. 1:**
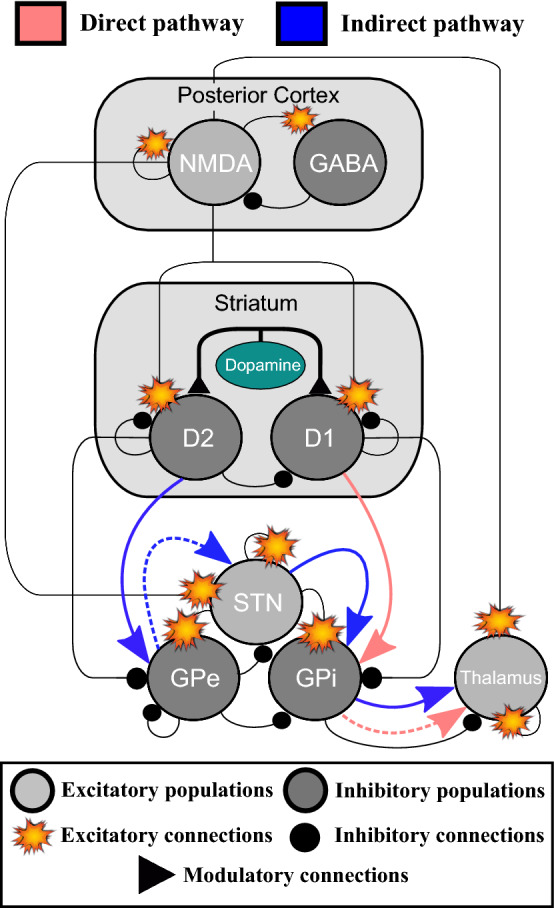
Diagram for the connections of the neuronal structures considered in the BG-thalamo-cortical model. The proposed model integrates the direct, indirect and hyperdirect pathways of the BG, in addition to some internal regulatory connections. The figure highlights the dominant activation of the direct (light red) and indirect (blue) pathways of the basal ganglia. The direct pathway is dominant if the level of dopamine is high enough. On the contrary, if the dopamine level is reduced, the indirect pathway is dominant. The inhibitory populations are in dark grey and the excitatory populations are in light grey. The connections with circles are inhibitory, the sparks show excitatory connections and the triangles show the modulatory effect of the dopamine input. The dashed lines represent a reduced signal flow as a consequence of the dopamine regulation. (Color figure online)

In the following sections, we will explain the single-neuron models for each neural population. Finally, we will present how the network is built by focusing on the interconnections between the cortex, the different nuclei of the BG and the thalamus. The connection diagram of the neural populations considered in our model is given in Fig. 1. In this figure, we also highlight the dominant activation of the direct and indirect pathways of the basal ganglia, which depends on the level of dopamine and will be explained in “Model of the striatum” and “Network model results” sections.

### Single-neuron models and results

Each individual neuron is modelled as a spiking point-like neuron of the threshold-firing type (Izhikevich 2003). That is: an auxiliary reset condition for the generation of the action potentials or spikes is considered in every single neuron. The parameters of each single neuron are determined by a phase-portrait analysis of the dynamical system’s response and the input-frequency relationships of the neurons in the corresponding brain regions. The advantage of the proposed method is that it captures essential dynamics with a simple enough model at single-cell level. Moreover, the reproduced spiking patterns give rise to experimentally-observed LFP oscillations which change as a consequence of the dopaminergic modulation at network level.

We will start by confirming the spiking patterns of the single-neuron models to match the empirically-validated data from reference studies. The single-neuron model parameters are obtained with the phase-portrait of the neuron dynamics as in Izhikevich (2007). Our methodology mainly depends on determining the number and stability properties of the system’s equilibrium points. The number of equilibrium points depends on the injected current, which may cause a bifurcation—the point which marks a change in behaviour. When the membrane potential of a neuron exceeds the firing threshold, the injected current reaches the rheobase current of the neuron. By analysing the system’s dynamics around the bifurcation point, we can exploit some of the experimentally-determined electrophysiological parameters of the single-neuron model, such as: the rheobase current, the rheobase voltage, the resting input resistance, and the membrane time constant. The rest of the parameters are chosen to provide similar spike widths, firing rates, and firing patterns to the ones reported in reference studies. For this purpose, we first examine the f-I response of the neurons to measure the frequency (f) of the spike generation for varying levels of the injected current (I). We then consider the spiking response of the neuron model by analysing the spike generation patterns around the rheobase current (frequency adaptation, initial spike latency, regular/irregular, or burst firing), in addition to the membrane potential response to hyperpolarising currents or changes in DA levels.

We highlight that the parameters used for each single neuron have been carefully chosen to ensure that every single neuron has the expected oscillatory behaviour. The membrane potentials for the single neurons in relevant populations of our model are provided in the following sections. Details of the equations and parameters used for the simulations are provided in the supplementary material to make our results reproducible.

#### Model of the striatum

The striatum is the primary input nucleus for cortical afferents to the BG (Albin et al. 1989). The cortical information is processed within the striatum and passed through the direct and indirect pathways to the output nuclei of the BG. Medium spiny neurons (MSNs) are the principal neurons within the striatum. They are GABAergic inhibitory cells and account for nearly the 95% of the total population of the striatal neurons (Kemp and Powell 1971). MSNs are the main target of the neurotransmitter dopamine. Dopamine tunes the excitability of the family of striatal-receptor subtypes. Our model considers two subtypes of MSNs: $D_1$- and $D_2$-receptor-type neurons. The assignment of these MSN subtypes is based on their axonal targets and are activated by G-type proteins, which excite ($D_1$-type) or inhibit ($D_2$-type) adenylyl cyclase (Nicola et al. 2000). This leads to opposite effects of the dopamine in the modulation of the excitability of the two main classes of the dopamine receptors, which in turn plays a key role in the regulation of the oscillatory activity within the BG.

The distinction between the response of MSNs to dopamine gives rise to two segregated pathways: the direct and indirect pathways. The balance between these pathways is regulated by the level of dopamine. We model this level of dopamine with a function $\phi (t)$, with values in [0, 1]. The level of dopamine can be adjusted to trigger the transitions between different BG states in the network model. To be more precise, in our model, we use two functions to reflect the level of dopamine: (1) $\phi _1(t)$, with values in [0, 1], for the model of $D_1$-MSNs, and (2) $\phi _2(t)$, with values in [0, 1], for the model of $D_2$-MSNs.

The function $\phi _1(t)$ at each time *t* expresses the proportion of active dopamine in the $D_1$ receptors. Values of $\phi _1$ close to 1 result in the over activation of $D_1$ receptors by modulating both the cortical input and membrane potential dynamics of $D_1$-MSNs. For the $D_2$ receptors, we use $\phi _2(t)$, which appears in the membrane potential dynamics for MSNs with $D_2$-type receptors. The higher the value of $\phi _2$ is, the more inhibition in $D_2$ receptors is produced. For the direct pathway’s dominance, $\phi _1$ has to be high enough, which means that the dopamine level is high enough to increase the activation of $D_1$-MSNs, and consequently, to activate the direct pathway. Moreover, $\phi _2$ has to be big enough to inhibit the population of $D_2$-MSNs. We will consider $\phi _1(t)=\phi _2(t)=\phi (t)$ for all *t*. This is explained in Sections 1.2.1 and 1.2.2. of the supplementary material attached to this paper.

An increase of the dopamine level, $\phi (t)$, allows the $D_1$-receptor-type neurons to enhance their neural response (direct pathway) while having an opposite effect on the $D_2$-receptor-type neurons (West and Grace 2002). A decrease in the dopamine level, $\phi (t)$, favours the dominance of the $D_2$-MSNs (dominance of the indirect pathway) over the $D_1$-MSNs. $D_1$-receptor-type neurons provide inhibitory feedback to the GPi so that the direct pathway is favoured. $D_2$-receptor-type neurons project onto the GPe for the indirect pathway to be dominant. The neural populations involved in these pathways are visualised for healthy activity of the BG network in Fig. 1.

**Fig. 2:**
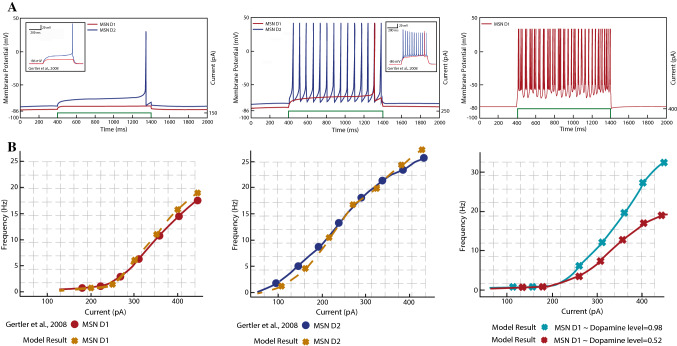
Results for the simulation of the single-cell dynamical model of $D_1$- and $D_2$-receptor-type MSNs. **A** Left: initial spike generation for $D_1$- and $D_2$-MSNs with $\phi =0.52$ and a direct current (DC) injection of 150*pA*. Middle: more activation of $D_2$-MSNs with $\phi =0.52$ and a DC of 250*pA*. The results are compatible with Gertler et al. (2008), the activity of both MSNs presents spike latencies for the first spike, and later, spikes occur without such a delay with L-type $Ca_2^+$ current contribution. Right: $D_1$-receptor-type MSN response to a DC of 400*pA* with a dopamine level of $\phi =0.98$. The green lines at the bottom of the figures show the strength of the injected current. **B** Left: f-I curve for the modelled $D_1$-receptor-type MSN compared to results in Gertler et al. (2008). Middle: f-I curve for the modelled $D_2$-receptor-type MSN compared to results in Gertler et al. (2008). Right: $D_1$-MSN model predicts the increase in the slope of the f-I curve with a dopamine level of $\phi =0.98$ and no change in the rheobase current

To consider all these features and by using data from a multi-compartment model (Gertler et al. 2008), we propose a dynamical model that is simple enough, yet electrophysiologically plausible, which can reproduce the key electrical properties of $D_1$- and $D_2$-receptor-type MSNs with different levels of dopamine. The simulated neuron’s firing behaviour and the f-I curve of both MSN subtypes are shown in Fig. 2. Our model is able to generate key spiking dynamics of MSNs: initial spike latency, dopamine-modulated state transitions, and enhanced sensitivity to a depolarising input during dopamine intervention (Gertler et al. 2008; Mahon et al. 2000; Nisenbaum et al. 1994). Without a stimulation, an MSN stays silent with a hyperpolarised membrane potential. When a low direct current (DC) is applied, the MSN shows spike latencies, and a sufficient cortical input is needed to cause a response.

Figure 2A shows the firing responses of both MSN subtype models, and how well the results fit with the multi-compartment model used as reference. In our model, the dopamine application is expressed by the dopamine function $\phi (t)$, which was explained above. For the simulation of single MSNs, we consider $\phi (t)$ as a constant $\phi$. We use two representative values for $\phi$: $\phi = 0.52$ and $\phi = 0.98$. The parameter values used for the simulations were derived from the models of single neurons that exhibited appropriate firing patterns and firing rates for each subtype of MSNs. The f-I curve of $D_1$-receptor-type MSNs—in Fig. 2B—shows a linear increase in the slope as the dopamine level is elevated (Moyer et al. 2007). When the dopamine level is high, the transition from the down-to-up state is more abrupt, and the up-state outlasts the dopamine-free condition. Once the rheobase voltage is achieved, the neuron fires with a shorter latency and a phasic burst-like doublet or triplet of spikes is present.

The striatum also includes fast-spiking interneurons (FSIs), which exhibit fast-spiking patterns (Centonze et al. 2003). Striatal FSIs represent local GABAergic interneurons. They form the main inhibitory input to the MSNs and provide a winner-takes-all mechanism for MSNs (Mallet et al. 2005). When the direct pathway is dominant over the indirect pathway, the FSIs’ GABAergic input to the $D_2$-MSNs is scaled up to favour the direct pathway. In a similar way, when the indirect pathway is dominant, the FSIs inhibit the activity of the $D_1$-receptor-type MSNs more than the activity of the $D_2$-receptor-type MSNs. With the goal of having the most simple model that can reproduce key dynamical global BG network behaviours, our model of the striatum does not include FSIs. The effect of FSIs on the MSNs is approximated by adequate parameters within the models for the MSNs. These parameters are linked to the regulation of dopamine. For details on how FSIs can be included within the striatum, the models proposed in Çelikok et al. (2016), Navarro-López et al. (2016) can be checked.

#### Model of the globus pallidus

The globus pallidus is subdivided by an internal medullary lamina with internal and external parts, which are called GPi and GPe (Telford and Vattoth 2014). There are two main routes that connect the striatum to the globus pallidus: (1) the direct pathway, comprising direct GABAergic projections to the GPi, and (2) the indirect pathway, comprising GABAergic projections to the external segment of the GPe. The GPe has widespread projections to the other BG nuclei. Consequently, the GABAergic control of the GPe plays a key role in the signal processing and modulation within the BG. The GPi works in an opposite way, in the sense that the GPi constitutes the primary output nucleus of the BG, and its ascending axons mostly innervate the thalamus.

When the direct pathway is dominant, the GPi receives inhibitory inputs from the striatal $D_1$-type MSNs. These inputs reduce the activity of the GPi and produce the excitation of the thalamus. The GPi receives an increased input from the STN when the indirect pathway is dominant, providing an extra excitation to the GPi neurons, which results in a reduction of the activity of the thalamus. The activity of the GPi is important for our model since it regulates the activity of the thalamus.

Both the GPe and the GPi are autonomous pacemakers capable of generating fast-spiking activity, even in the absence of excitatory inputs (Mercer et al. 2007). The main difference between the firing patterns of the GPe and the GPi neurons is that the GPi neurons show a higher frequency activity (DeLong 1971). Although both pallidal segments share similar morphology and neurotransmitter –c-aminobutyric acid (GABA)–, they project to different BG pathways (Nambu 2007). Another difference between the GPe and the GPi populations is in the properties of their local axonal collaterals that terminate on neighbouring cells. GPe neurons have a rich amount of internal connections to other GPe neurons, whilst GPi neurons do not have a significant amount of internal connections with other GPi neurons (Parent and Hazrati 1995).

**Fig. 3:**
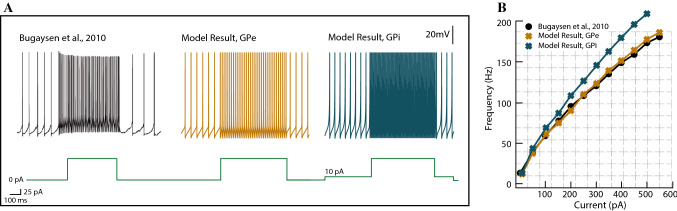
Results for the simulation of the single-cell dynamical model of the GPe and the GPi. **A** The GPe neuron model responds to a depolarising input with high-frequency spikes. Spontaneously, the active cell increases its firing in response to a depolarisation in a regular manner. The GPi neuron model is tuned to be slightly more sensitive to a depolarising input. The green lines at the bottom of the figures show the strength of the injected current. **B** The GPe neuron model is tuned to mimic the GPe cell recordings of Bugaysen et al. (2010). (Color figure online)

From an electrophysiological viewpoint, the GPe consists of two subpopulations of neurons with different firing patterns: the high-frequency pausers (HFP) (85%) and the low-frequency bursters (15%) (Bugaysen et al. 2010). Here, we consider the GPe as a homogeneous structure of HFP neurons that works as a mere relay device in the indirect pathway. The HFP neurons of the GPe are GABAergic and spontaneously active, and are able to generate high-frequency and irregular spikes in vivo (Bugaysen et al. 2010). The irregularity in the HFP firing occurs because the high-frequency regular spiking activity is interrupted by pauses. We do not include this feature in our single-cell model. However, we characterise this pause-generating process as a synaptically-driven event. Like GPe neurons do, GPi neurons fire spontaneously at high frequencies. Unlike the GPe, the GPi fires without pauses (DeLong et al. 1985). With this in mind, we consider the electrophysiological properties of both pallidal neurons similar, but with a slightly higher basal firing rate for the GPi neuron. Our model is able to generate high-frequency spikes for the GPe neuron by adjusting the spike-width as offered in Bugaysen et al. (2010). The spiking response for the GPe and the GPi neuron models with different levels of the applied current and the f-I response curve are given in Fig. 3.

#### Model of the subthalamic nucleus

The STN has been typically considered as no more than a relay serving as another gate for ascending BG axons. However, there are strong evidences for the STN to be one of the main regulators of motor function related to the BG (DeLong et al. 1985). The fact that the STN is the only excitatory structure within the BG reinforces this key regulation role. Given its anatomically central position and the fact that it targets different BG nuclei, the STN is likely to play a key role in the BG. From a clinical viewpoint, this is also evident, considering the involvement of the STN in movement disorders such as Parkinson’s disease (DeLong 1990). Another key feature of the STN is that it receives direct inputs from the cortex, bypassing the striatum.

**Fig. 4:**
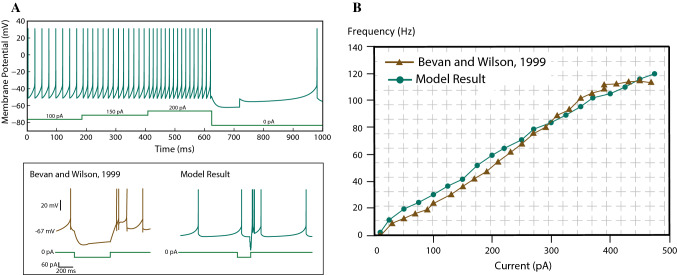
Results for the simulation of the single-cell dynamical model of the STN. **A** Modelled cell responses to depolarising and hyperpolarising inputs. Up: the model responds to a depolarising input with an increased frequency; there is also an spontaneous activity without any stimulation. Bottom: a hyperpolarisation causes a rebound of burst spikes followed by a spontaneous low-frequency firing state. The green lines at the bottom of the figures show the strength of the injected current. **B** The frequency response of the model fits well with the results of the experiments given in Bevan and Wilson (1999). The slope of the f-I curve slightly increases around 30 Hz. (Color figure online)

The STN receives its major afferents from the cerebral cortex and the GPe, and projects mainly to both segments of the globus pallidus (GPe, GPi). In a healthy brain, there are three main different firing patterns of an STN neuron (Bevan and Wilson 1999). In the absence of synaptic stimulation, the cells of the STN fire spontaneously. However, by increased depolarisation, the STN cells are capable of transiently firing at high frequencies (Bevan and Wilson 1999). A nonlinearity is also observed in STN neuron’s membrane dynamics in *in vivo* recordings, suggesting that when the membrane potential of an STN cell is hyperpolarised below $-75$mV, it can be transiently depolarised through a hyperpolarisation-activated sag current (Wichmann et al. 1994). As a result, the STN cells are capable of generating rebound spikes as response to hyperpolarising currents. Our proposed STN model exhibits a slightly sigmoidal frequency-current relationship with the steeper portion starting around 40 Hz as proposed in Bevan and Wilson (1999). However, we do not consider the ionic mechanism giving rise to such a nonlinearity. This property increases the sensitivity of the STN neuron to high-frequencies associated with movement. The spiking response of the STN neuron model with different levels of the applied current and the f-I response curve are given in Fig. 4.

#### Model of the posterior cortex

We consider the posterior cortex, where the sensorimotor input –represented as an external input—arrives. In the last decade, a significant number of computational neuroscience studies have demonstrated that simplified spiking-neuron models of cortical circuits are capable of reproducing a wide range of cortical computations and firing patterns (Ainsworth et al. 2012; Koepsell et al. 2010). Excitatory and inhibitory reciprocal interactions generate cortical rhythmic-oscillations, and a simplified network model can be employed to study the mechanisms of neural oscillations, with implications in various neural information processes (Stefanescu and Jirsa 2008). Our model of the posterior cortex consists of two neural populations: excitatory neurons (glutamatergic with NMDA receptors) of the regular-spiking type, and inhibitory neurons (GABAergic) of the fast-spiking type.

Glutamate and GABA are considered the main excitatory and inhibitory neurotransmitters of the cortex. The excitatory NMDA receptors for glutamate stay inactive at their membrane potentials. Ion channels are mostly blocked and prevent positively charged ions from flowing in. An incoming signal from a pre-synaptic unit releases the inhibition on the ion channels, which excite the post-synaptic neurons. In contrast, GABAergic neurons use inhibitory neurotransmitters for communication. They show fast responses to inputs. Fast-spiking activity allows them to have a sufficient effect on NMDA receptors excitability (Petroff 2002). They work as a regulatory unit within the cortical populations and only communicate with neurons within the cortex. The balance between excitation and inhibition is crucial to maintain proper cortical function.

In our model, excitatory neurons of the posterior cortex show regular-spiking activity because most of the neurons in the cortex have regular-spiking behaviour. When they receive a prolonged stimulus, the neurons produce some spikes with short inter-spike period (Izhikevich 2003). During the following stimulation, these neurons show a reduction in the firing frequency of their spike response (Benda and Herz 2003). This is called the spike-frequency adaptation. These neurons have large spike-after-hyperpolarisations. Consequently, even if the injected current increases, a fast-spiking behaviour cannot be observed. Inhibitory GABAergic neurons of the posterior cortex generate low-threshold spikes (LTS). These neurons can produce high-frequency trains of action potentials (Izhikevich 2003). Similarly to regular-spiking neurons of the cortex, LTS neurons exhibit spike-frequency adaptation. They have a low spike threshold, which enables them to generate spikes with high frequencies, but they may also generate instability. That is, depending on the membrane potential and the current input, they may also exhibit regular, irregular, and burst firing (Beatty et al. 2012). LTS neurons have higher depolarised resting potentials and lower input resistances than regular-spiking neurons (Izhikevich 2007). Inhibition by LTS neurons prevent cortical neural populations from getting overexcited.

**Fig. 5:**
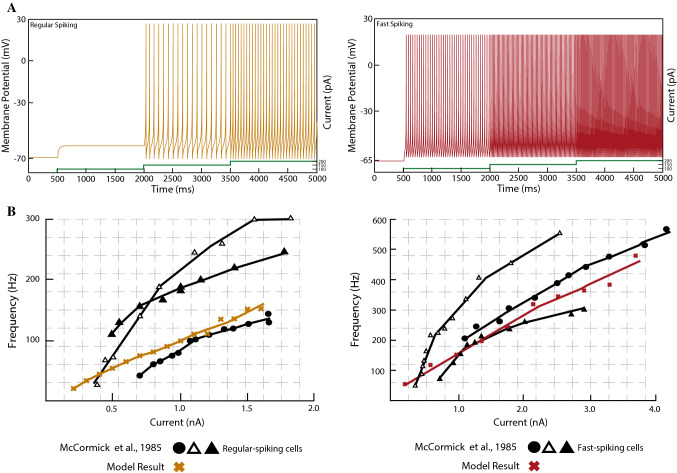
Results for the simulation of the single-neuron dynamical models of the cortical regular-spiking neurons (NMDA) and the fast-spiking neurons (GABAergic). **A** Responses for the modelled neurons to a depolarising input. Left: regular-spiking cortical neuron. Right: fast-spiking cortical neuron responds to a depolarising input with an increased frequency; a spike-frequency adaptation is observed for the initial spikes. The green lines at the bottom of the figures show the strength of the injected current. **B** f-I curves for the regular-spiking neuron (Left) and the fast-spiking (Right) neuron models compared to the recordings from McCormick et al. (1985). (Color figure online)

Figure 5 shows the spiking response of both cortical neuron models with different levels of the applied current and the f-I response curve. We reproduce satisfactorily the regular spiking activity of cortical excitatory neurons, which fires tonic spikes with a decreasing frequency (Izhikevich 2003). That is, the frequency is relatively high at the onset of some stimulation, and then it adapts. Fast-spiking inhibitory neurons respond in a similar way to regular-spiking excitatory neurons, but they exhibit a higher frequency response to a depolarising input (McCormick et al. 1985).

#### Model of thalamocortical relay neurons

The thalamus is a centrally-located brain structure that is considered to act as a hub that receives information from subcortical areas and projects onto the primary cortical areas associated with motor actions and cognitive functions (Sherman and Guillery 2002). Anatomically, there are three different types of neurons within the thalamus: thalamocortical (TC) neurons—which relay signals into the cortex–, reticular thalamic nucleus (RTN) neurons, and thalamic interneurons. We only consider thalamocortical (TC) relay neurons for the sake of simplicity. TC neurons play a key role in PD.

Thalamic cells must be modelled to show tonic activity when presented with a low-amplitude input and a bursting activity in the presence of a high enough input (Deschenes et al. 1984). One key spiking property of TC neurons is the transition between the tonic and burst firing modes, where the transition primarily depends on the level of the inactivation of a low-threshold T-type calcium ($Ca_2^+$) current (Zhan et al. 1999). As the cell is hyperpolarised to a certain level (− 80 mV, for example), the T-type $Ca_2^+$ channel, first deactivates, and then, becomes fully activated during the repolarisation of the membrane. This process makes the thalamic neuron generate $Ca_2^+$ spikes in rebound bursts. In the tonic model, the neuron generates sodium-potassium ($Na^+$–$K^+$) spikes that are more regular, but they may also display frequency adaptation (Zhan et al. 1999). In our model, we take into account the dynamics of $Ca_2^+$ and $K^+$ currents in a simplified manner in order to achieve desired firing properties of TC neurons.

**Fig. 6:**
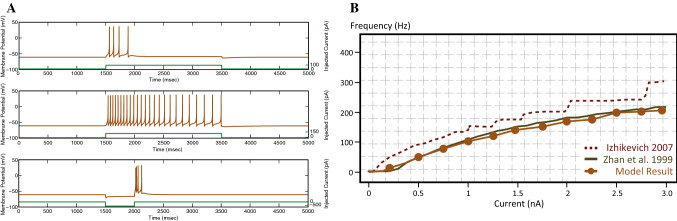
Results for the simulation of the single-cell dynamical model for a TC relay neuron. **A** The TC neuron model mimics three essential features of the firing pattern of TC relay neurons: (1) initial burst spikes when injected with current just above the rheobase level, (2) tonic spiking with increased current injection, and (3) rebound burst in response to hyperpolarisation. The green lines at the bottom of the figures show the strength of the injected current. **B** The frequency response of our TC neuron model well matches the experimental data in Zhan et al. (1999). (Color figure online)

The spiking response for our TC neuron model with different levels of the applied current and the f-I response curve are given in Fig. 6. An injected current above the rheobase level is able to evoke a single burst firing. A larger current injection is required for the neuron to remain active. As the applied current increases, the neuron generates repetitive and regular spikes, after an initial burst with frequency adaptation (Zhan et al. 1999). When the neuron is hyperpolarised below − 80 mV for 500 ms, there is an excess of $Ca_2^+$ currents. Moreover, when the hyperpolarisation is relieved, the membrane potential overshoots the firing threshold, resulting in an interval of high-frequency rebound spikes (Zhan et al. 1999). For the thalamic cells, we employed an expanded version of the model proposed by Izhikevich (2007), with an additional second slow variable. Our modification gives a better quantitative measure with the input-frequency response of TC recordings from Zhan et al. (1999). The details are in the supplementary material.

### Building the network from single neurons

The connection diagram of the network model which integrates the neural populations described above can be seen in Fig. 1. We are mainly focused on the analysis of the direct and indirect pathways of the BG and their relationships with some PD scenarios. Although we do not directly associate the hyperdirect pathway of the BG with specific PD-related processes, we consider the connections which form the hyperdirect pathway as important internal regulatory mechanism within the proposed network model. The hyperdirect pathway bypasses the striatum, and connects the cortex to the GPi via the STN. The hyperdirect pathway is critical for suppressing erroneous movements. When it fails, patients are unable to inhibit unwanted motor patterns and cause involuntary movements, which results in a condition known as hemiballismus (Postuma and Lang 2003). The message of the hyperdirect pathway can be translated as ‘hold your horses’ as it is suggested in Frank (2006).

In our BG network model, the neuron dynamics define the evolution of each neuron population’s behaviour over time, and determine the way neurons behave according to inputs. In contrast, the connections between neurons within a population and between populations of neurons represent the evolution of the relationships between them. These adaptive neuronal processes are generally known as neuroplasticity. This evolution depends on the firing activity and the firing times of the neurons.

We expect that starting from randomly-assigned connections and synaptic strengths, the connections between populations evolve into a state where the model is capable of showing different emergent behaviours. That is why the connections between neurons are evolving connections. The idea is to model how local changes in the connection (synapse) between two neurons can affect the network dynamics to produce a self-organised global behaviour that did not exist in the neuron’s local behaviour.

In this context, an existing connection between two neurons may disappear (the synaptic strength becomes 0) or a non-existent connection may be formed (the synaptic strength becomes positive or negative) depending on the spike times. With this, our model can capture what is known as *structural plasticity* (Lamprecht and LeDoux 2004). That is, not only is synaptic behaviour modified, but synapses may also be rewired. This concept of structural plasticity can be also associated to changes in the network topology, which is also termed *wiring plasticity* (Chklovskii et al. 2004).

We will use a simplified model for the synaptic plasticity: the most simple model that is capable to reproduce key network states related to PD. With this in mind, evolving synaptic plasticity is only considered for the connections within the cortical NMDA neurons, the connections between the cortical NMDA and GABAergic neurons, and finally, for the connections between the cortical NMDA neurons and the striatal $D_1$-MSNs. We target these populations to focus on the effect of dopamine levels on PD. The plasticity of the synapses for these connections is modelled by a spike-timing-dependent plasticity (STDP) model. With this model, we will implement a global reward learning for the system to code action selection and recognise the effects of the applied stimuli in the cortex. In our model, the STDP properties of intra-cortical and cortico-striatal synapses differ from each other as indicated by empirically-validated studies (Fernando et al. 2008; Gurney et al. 2015). The rest of the connections within the network are arranged to have fixed synaptic strengths. Additionally, we limit the values of the synaptic strengths in order to avoid instabilities in the simulation. This is the most simple option. Other stabilisation or control mechanisms should be considered (like synaptic scaling) in a more sophisticated STDP model. The details of the STDP model and the connections between neurons, with all the parameters used, are provided in the supplementary material.

For the simulations, we consider the following set up. As established in “Model of the posterior cortex” section, the cortical network consists of two different types of subpopulations: excitatory and inhibitory. We use the ratio of excitatory-to-inhibitory number of neurons of 4:1 (800:200 neurons). This ratio is determined by the anatomy of a mammalian cortex (Noback et al. 2005). In the striatum, we consider 200 striatal $D_1$-MSNs and 200 $D_2$-MSNs. For the rest of the neural populations, we consider 100 neurons. All the neurons in the model are connected according to a random graph, and the initial probabilities in the local connections within a population, and the interconnections between neurons of different populations, are chosen in accordance to neurophysiological studies reported in the literature. Some of the connection probabilities are chosen to be higher to allow the pre-synaptic population to drive the post-synaptic population more accurately. We also make some of the inhibitory synaptic couplings stronger than some of the excitatory ones to allow the inhibitory neurons to adequately regulate the excitatory populations behaviour.

We also consider conduction delays between and within each neural population. Conduction delays reflect axonal distances between connected neurons and populations, and allow each population to have different synchronisation features. These data have been extracted from experimental studies reported in the literature, and the details are given in the supplementary material.

We highlight that the DA levels have a dual role in the network model (Bergman et al. 2015; Schultz et al. 1997). First, the DA interferes with the excitability of the MSNs subtypes in the BG network. Second, the DA alters the efficacy of the cortico-striatal synapses acting on both cortex-to-$D_1$ MSNs and cortex-to-$D_2$ MSNs. To model the DA influence in the BG network, we consider two different parameters. The first parameter, $\phi _{tonic}$, indicates the tonic DA level, which defines the basal level of the DA in the circuit. The second DA parameter, $\phi _{phasic}$, determines the phasic DA release when a sensory/motor input is required to be delivered to the thalamic network. A sudden increase in the phasic DA level allows the transmission of this information by channeling it through parallel BG pathways. In our model, we consider $\phi _{tonic}\ge 0$ and $\phi _{phasic} \ge 0$ such that the sum of $\phi _{tonic}+\phi _{phasic} \in [0,1]$. This is explained in the supplementary material.

Finally, in order to ensure that each local network has an appropriate basal firing rate in the absence of a dopaminergic or external interference, we additionally provide external Poisson-type spike trains to each neuron to compensate the part of the missing external inputs (Galvan et al. 2015).

## Network model results

The model of the BG-thalamo-cortical network is simulated under two different protocols: (1) non-pathological or healthy states without PD, and (2) states compatible with PD. Five representative scenarios will be presented in this paper: three are non-pathological and two are compatible with PD. For these five scenarios, the DA level is modelled as a time-varying function $\phi (t)$; the expression is given in the supplementary material (Section 1.2.1).

The non-pathological scenarios correspond to the situation where the DA levels over the network lead to network behaviours within a healthy range. We test the network model for three different non-pathological or healthy cases:

1. *Default network mode*. “Default network mode” section reports the first non-pathological or healthy scenario when no external stimulus is injected to the cortical neural populations. The DA level is only determined by a tonic DA level as the cortex is not stimulated. We consider $\phi (t)=\phi _{tonic}=0.5$ for all *t*. The low DA level allows the BG nuclei to display appropriate LFP activity during a resting network state, where there is no external intervention to the cortical network and we have a low DA release within the striatum, and hence, the network is at basal levels.
2. *Healthy behaviour: adequate response to external stimulus with a high enough dopamine level to make the BG direct pathway dominant*. “Action selection pathway enabled” section shows the second healthy scenario. The cortical populations receive an external stimulus, and an adequate DA release enables the BG to convey the cortical information to the thalamus during action selection. An adequate DA level is provided with the transient phasic DA level (with $\phi _{phasic}=0.5$), which is added to the tonic DA level ($\phi _{tonic}=0.5$). This cortical information is conveyed to the thalamus by both modulating the strength of the cortico-striatal synapses and altering the excitability of the striatal MSNs by the DA release. This scenario corresponds to the case where the action-selection BG direct pathway is enabled by the dopaminergic regulation. That is, the ‘BG gate’ opens for information transmission. For the simulation of the dopamine level $\phi (t)$, we consider equation (13) of the supplementary material with $\phi _{tonic}=0.5, \phi _{phasic}=0.5$.
3. *Healthy behaviour: adequate response to external stimulus with a low dopamine level to make the BG indirect pathway dominant*. “Action selection pathway disabled” section analyses the third healthy case. We test the capability of the network model in preventing the cortical activity from reaching the thalamus when there is no phasic DA release ($\phi _{phasic}=0$), but still the cortical network is activated with an external input. Even though there is no phasic DA release, this case corresponds to a ‘healthy’ behaviour and the DA level is determined by the tonic DA level ($\phi _{tonic}=0.5$). The difference between this case and case 1) (default network mode) is that in 3) there is cortical stimulation. This case reproduces the inhibition of a competing input through the BG-thalamic loop, allowing the transient selection of another competing input signal by the striatum. This corresponds to the situation when the BG indirect pathway predominates over the direct pathway, and the BG channel is blocked for information transfer. For the simulation of the dopamine level $\phi (t)$, we consider equation (13) of the supplementary material with $\phi _{tonic}=0.5, \phi _{phasic}=0$.

For the scenarios compatible with PD, the DA level is reduced to mimic the dysfunctional behaviour of DA-producing neurons. For the simulations, we reproduce the changes in the LFP activity and firing rates of the BG nuclei for two cases:

4. *Activity compatible with PD: inadequate response to external stimulus when the BG direct pathway is dominant*. “Action selection pathway enabled compatible with Parkinson’s disease” section reports the first PD-related state. We test the model’s selection capabilities when the phasic DA level is reduced in comparison to the healthy scenario 2) with $\phi _{phasic}=0.4$. The tonic DA level ($\phi _{tonic}=0.5$) is the same that was considered in scenario 2). The phasic DA level attains its highest value whenever the cortex is stimulated and decays with time when the cortical stimulus stops. The purpose of this case is to confirm how the BG communication is impaired and how the BG network is incapable of appropriately conveying cortical signals to the thalamus. For the simulation of the dopamine level $\phi (t)$, we consider equation (13) of the supplementary material with $\phi _{tonic}=0.5, \phi _{phasic}=0.4$.
5. *Activity compatible with resting tremors in PD*. “Default network mode compatible with Parkinson’s disease” section evaluates the network activity in the PD ‘resting’ state when no external stimulus is injected to the cortical network, consequently, in this case, there is no phasic DA level and the tonic DA level is the lowest one. That is, we have $\phi (t)=\phi _{tonic}=0$ for all *t*. The consideration of this case is valuable in order to compare the alterations present in the PD resting-sate network activity with the default network mode of scenario 1).

We highlight that our neural network model is a qualitative representation of reality which is biophysically realistic, as it was demonstrated in “A dynamical model of the basal ganglia-thalamo-cortical network” section. In this sense, the values for the dopamine level are relative, and the reduction or increase of dopamine values is more relevant than the absolute values.

Twenty simulations were completed for each of the five scenarios with different randomly-connected networks. The overall activity rate of the last 7 s of 10-min-long simulations were analysed. This is to consider a stabilised activity of the network.

We will present the power spectral analysis for each neural population in order to show that the results obtained with the proposed model are qualitatively in agreement with already-published experimental results that are explained throughout the paper, especially in the Introduction and “A dynamical model of the basal ganglia-thalamo-cortical network” section. We will visualise key features of our spectral analysis with spectrograms and power histograms of frequencies. These are obtained from the firing rates for each population of neurons and following these steps for each neural population:

1. *Number of neurons that fire at time t*. At each time *t* of the simulation, the number of the neuron that has fired is recorded and represented in a raster plot. Every dot in the raster plot corresponds to a spike of the corresponding neuron of the population at a given time *t*.
2. *Average firing rate*. In order to give a statistically-meaningful result, the simulation of our network model is repeated twenty times, and the average of the firing rate for each population is obtained. For this, we use the information of each raster plot and calculate the number of neurons that fire simultaneously (average number of spikes) within a time window *T*. We add up the number of spikes within the window *T* and divide it by *T* to obtain the firing rate in hertzs (Hz). Our time window is $T=50$ ms. We repeat this computation twenty times and obtain the average firing rate for each neural population.
3. *Frequency analysis*. The average firing rate obtained for each neural population in Step 2 is used to carry out our frequency analysis. The power spectral analysis, which is summed up with the spectograms and power histograms of frequencies in the figures, is obtained with the MATLAB-script LFP Analysis Suite developed in the Neural Networks Laboratory of the University of Nottingham (Peter and Aldam 2006). The LFP Analysis Suite makes use of the first principal component analysis (PCA) to sort out the spikes, and the time series corresponding to the spike trains are modelled with the vector autoregressive model (VAR). The partial directed coherence (PDC) and the generalised PDC are considered to obtained the power spectral analysis (Taxidis et al. 2010). The LFP Analysis Suite has been typically used for data collected from LFP recordings and single-unit activities, however, we apply it to the firing rates obtained as a result of the simulations of our model.

In the figures presented in the following sections, while each raster plot represents one of many simulations, the spectrograms and power histograms are obtained from the average firing rates of twenty simulations.

In this study, we consider the nature of firing rates and LFP in the BG, how these features are modulated through the BG internal communication, and finally we look at their influence over the thalamus. All our hypotheses and the design of the simulations are based on already-published experimental studies of rats, monkeys with (1-methyl-4-phenyl-1,2,3,6-tetrahydropyridine) MPTP or (6-hydroxydopamine) 6-OHDA treatment, and PD human patients, as reference for the changes in the BG activity due to PD-related alterations (see the Introduction for details). All the equations and parameters used to reproduce these scenarios are presented in detail in the supplementary material.

### Default network mode

By analysing the network activity with no external stimulus, we can better understand how the transition from the resting state to any other state is manifested in the changes of the activity of the different neural populations. The network activity with no external stimulus and no phasic DA intervention is what we refer to as the default mode or resting state of the network. The dopamine level is determined by a healthy tonic level.

**Fig. 7:**
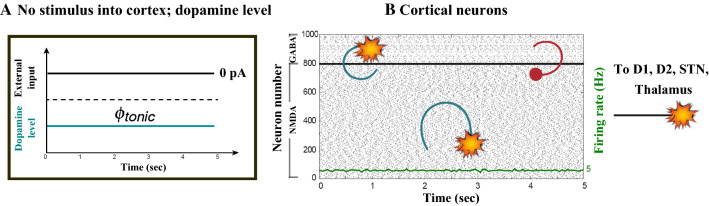
Default network mode. **A** No external stimulus is applied to the cortical neural populations (NMDA and GABA-receptor populations) and the level of dopamine is defined by $\phi (t)=\phi _{tonic}=0.5$ for all *t*. **B** Raster plot of the spiking activity of the simulated populations of the cortical neurons when no external stimulus is injected to the cortical network and the level of dopamine is defined by $\phi _{tonic}=0.5$. The NMDA-cortical population’s excitatory response is applied to the $D_1$-MSNs, the $D_2$-MSNs, the STN and the thalamus, and the result is given in Fig. 8. Each black dot corresponds to a spike of the corresponding neuron at a given time. The green function on the raster plot shows the average firing rate of the NMDA-cortical neurons for the 20 simulations produced. The raster plot is for one of these simulations. The sparks indicate excitatory connections, whereas the circles are inhibitory connections

**Fig. 8:**
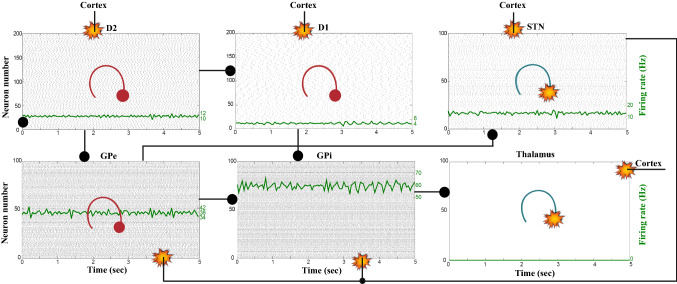
Default network mode. Raster plots of the spiking activity of the relevant simulated neural populations when no external stimulus is injected to the cortical network and the level of dopamine is defined by $\phi (t)=\phi _{tonic}=0.5$ for all *t*. The associated dopamine level, the external stimulus applied to the cortex and the NMDA-cortical neurons’ response applied to the $D_1$-MSNs, the $D_2$-MSNs, the STN and the thalamus are given in Fig. 7. Each black dot corresponds to a spike of the corresponding neuron at a given time. For each population, the green functions on the raster plots show the average firing rate for the 20 simulations produced. The raster plots are for one of these simulations. The sparks indicate excitatory connections, whereas the circles are inhibitory connections

**Fig. 9:**
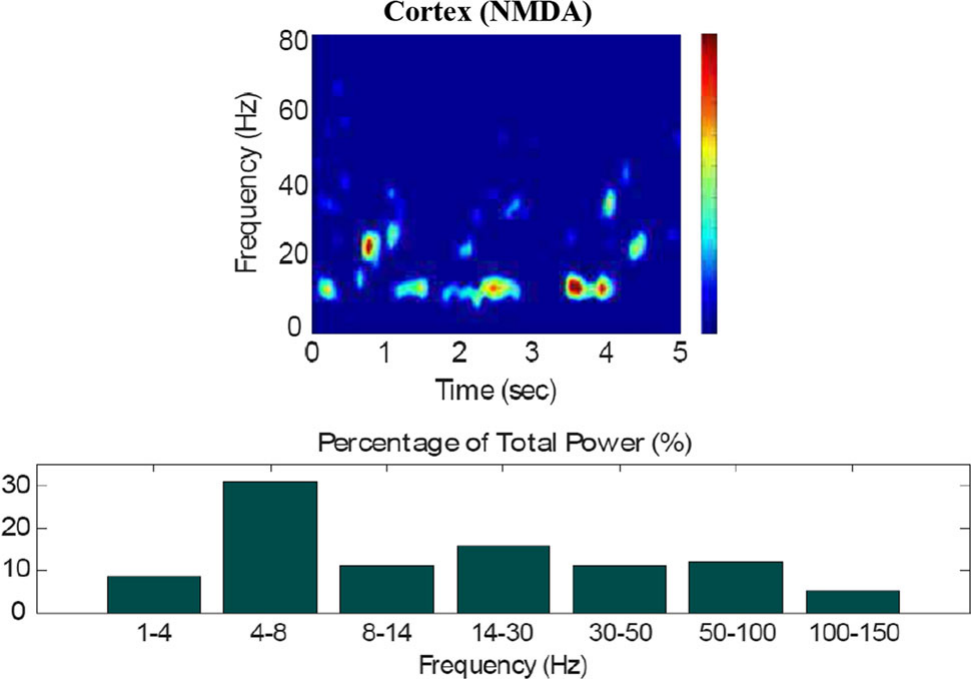
Default network mode. Spectrogram and power histogram analysis results of dominant frequencies for the firing activity of the NMDA-cortical neural population

**Fig. 10:**
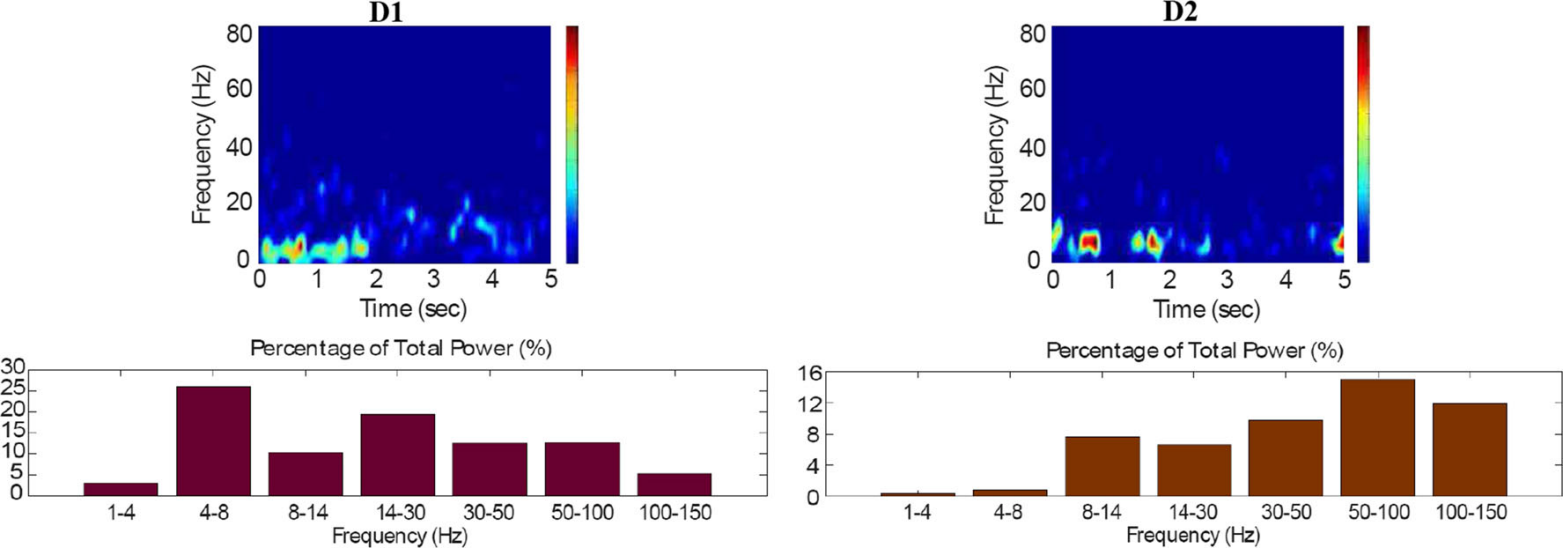
Default network mode. Spectrogram and power histogram analysis results of dominant frequencies for the firing activity of the $D_1$- and the $D_2$-MSNs of the striatum

**Fig. 11:**
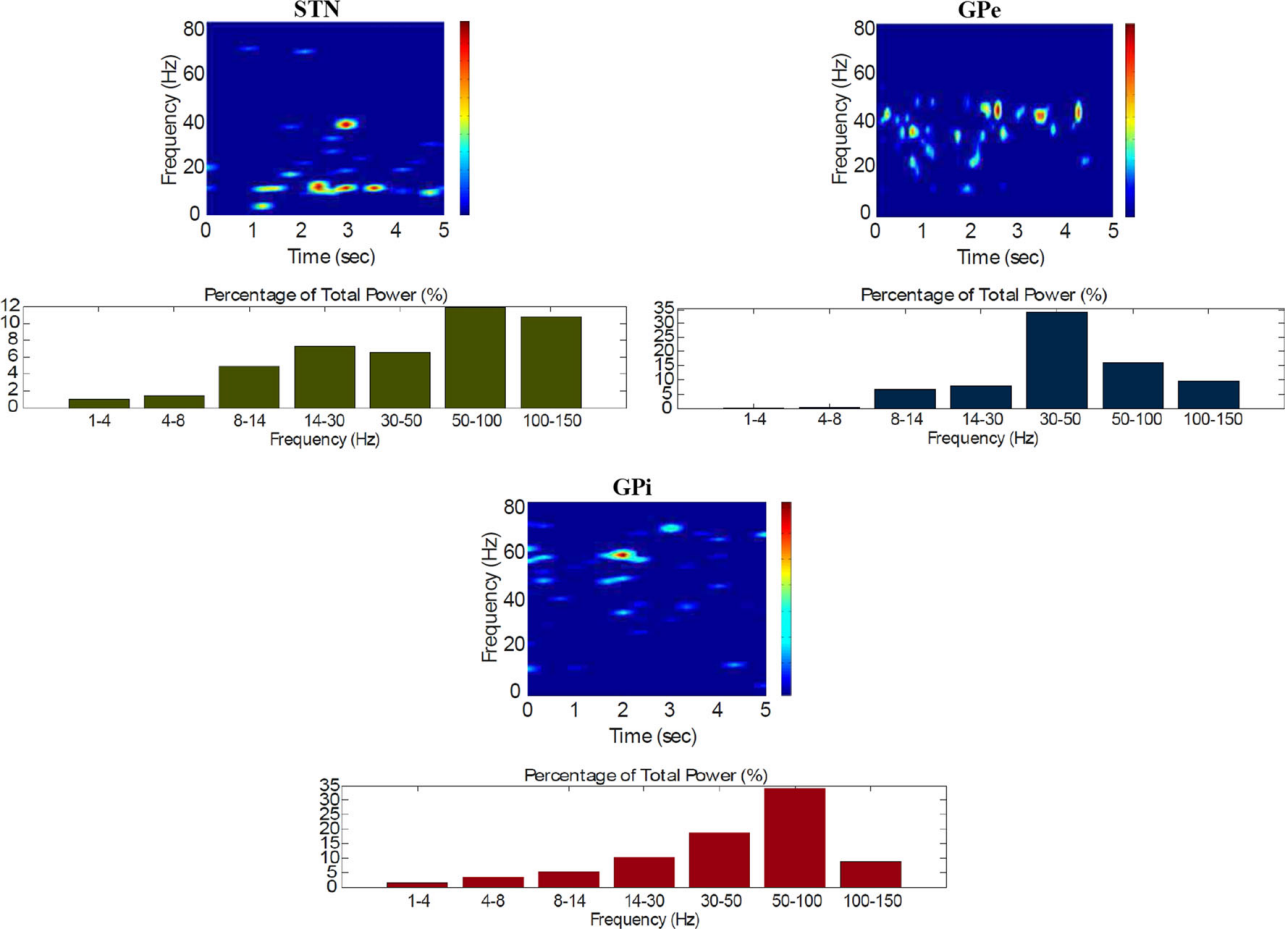
Default network mode. Spectrogram and power histogram analysis results of dominant frequencies for the firing activity of the STN, the GPe and the GPi

Figures 7, 8, 9, 10 and 11 show the raster plots and frequency analysis of the spiking activity of the main populations of neurons in the default network mode. What we can appreciate from the figures is that the posterior cortex (Figs. 7, 9) shows a spontaneous and low-frequency activity that is not capable of providing enough excitation to the MSNs striatal populations (Figs. 8, 10). There is a prominent theta-frequency band activity (4–8 Hz) in the cortical and $D_1$-MSN network.

However, the $D_2$-MSN and STN populations fire at a higher basal firing rate with a frequency range close to an upper boundary of the alpha-frequency band (8–13 Hz), as it is shown in Figs. 10 and 11. In this model, a high-frequency activity of both GPe and GPi neurons exerts a constant inhibitory influence on their efferent pathways (Figs. 8, 11). They do so to provide, precisely, timed pauses for thalamic disinhibition to encode information when pause timings are controlled by DA triggering. As a result, the thalamic network remains silent through the entire simulation.

The power spectral densities provide similar results to the firing rates. However, the power spectral densities of the GPe, $D_2$-MSNs, and the thalamus contain more power in higher frequency ranges; this is probably due to large-amplitude subthreshold oscillations at higher frequencies. Another important conclusion can be drawn from the frequency analysis in Figs. 10 and 11: none of the populations display their prominent activity at beta-frequency band (13–30 Hz), and the local network activities of each BG population were obtained to be uncorrelated. This is considerably important since these features will be used as a measure of PD in later simulations. The overall mean firing rates and the frequency responses obtained with our model are qualitatively in good agreement with experimental results (see (Obeso et al. 2008) and references therein for more details). Additional references are given in the Introduction.

### Action selection pathway enabled

**Fig. 12:**
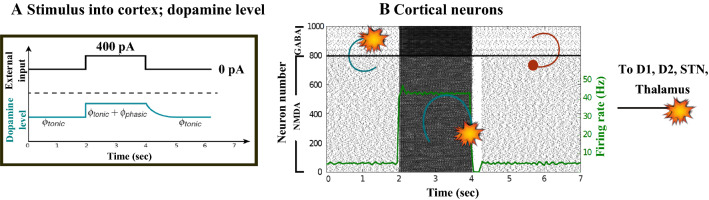
Healthy behaviour: adequate response to external stimulus with a high enough dopamine level ($\phi _{tonic}=0.5$ and $\phi _{phasic}=0.5$) to make the BG direct pathway dominant. **A** External stimulus applied to the cortical neural populations (NMDA and GABA-receptor populations) and dopamine level, $\phi (t)$, increasing with cortical stimulation. **B** Raster plot of the spiking activity of the simulated populations of the cortical neurons when the action selection (direct) pathway is enabled by an adequate level of dopamine while an external stimulus is applied into the cortex. The NMDA-cortex population’s excitatory response is applied to the $D_1$-MSNs, the $D_2$-MSNs, the STN and the thalamus, and the result is given in Fig. 13. Each black dot corresponds to a spike of the corresponding neuron at a given time. The green function on the raster plot shows the average firing rate of the NMDA-cortical neurons for the 20 simulations produced. The raster plot is for one of these simulations. The sparks indicate excitatory connections, whereas the circles are inhibitory connections

**Fig. 13:**
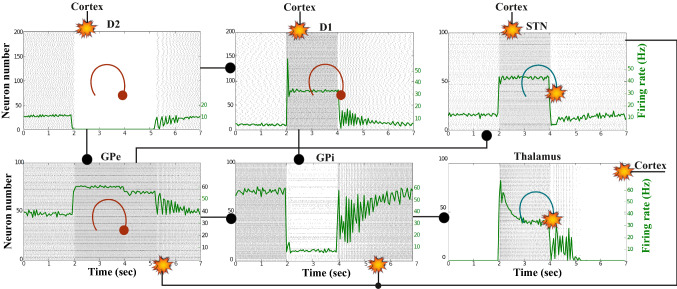
Healthy behaviour: adequate response to external stimulus with a high enough dopamine level ($\phi _{tonic}=0.5$ and $\phi _{phasic}=0.5$) to make the BG direct pathway dominant. Raster plots of the spiking activity of the relevant simulated populations of neurons when the BG action selection (direct) pathway is enabled by an adequate dopamine level while an external stimulus is applied into the cortex. The associated dopamine level, the external stimulus and the NMDA-cortical neurons’ response applied to the $D_1$-MSNs, the $D_2$-MSNs, the STN and the thalamus are given in Fig. 12. Each black dot corresponds to a spike of the corresponding neuron at a given time. For each population, the green functions on the raster plots show the average firing rate for the 20 simulations produced. The raster plots are for one of these simulations. The sparks indicate excitatory connections, whereas the circles are inhibitory connections. The DA release causes the depolarisation of the $D_1$-MSNs and the hyperpolarisation of the $D_2$-MSNs. The inhibition of the $D_1$-MSNs in the striatum flows directly to the GPi. In turn, the GPi releases inhibition over the thalamic neurons

**Fig. 14:**
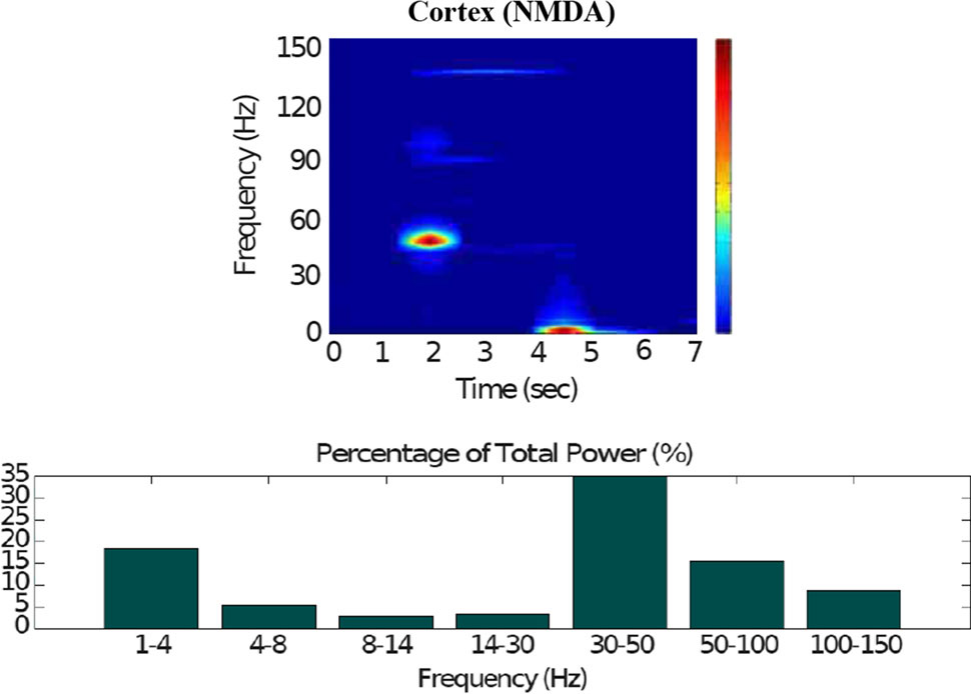
Healthy behaviour: adequate response to external stimulus with a high enough dopamine level to make the BG direct pathway dominant. Spectrogram and power histogram analysis results of dominant frequencies for the firing activity of the NMDA-cortical neural population

**Fig. 15:**
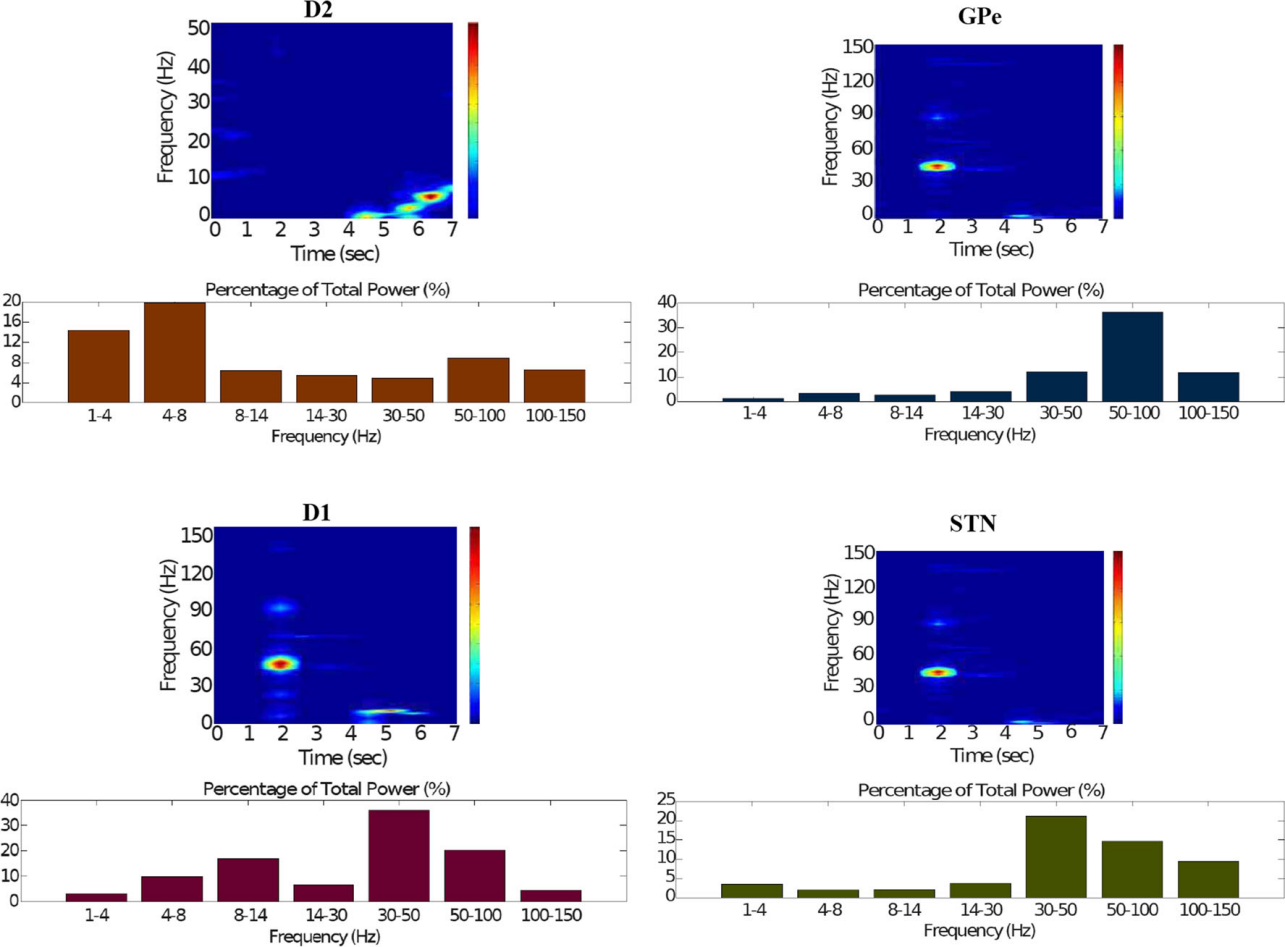
Healthy behaviour: adequate response to external stimulus with a high enough dopamine level to make the BG direct pathway dominant. Spectrogram and power histogram analysis results of dominant frequencies for the firing activity of the $D_2$-MSNs, the GPe, the $D_1$-MSNs and the STN. The dominant frequencies of the STN-GPe network are at gamma-frequency band (30–150 Hz) and the network synchronisation is low

**Fig. 16:**
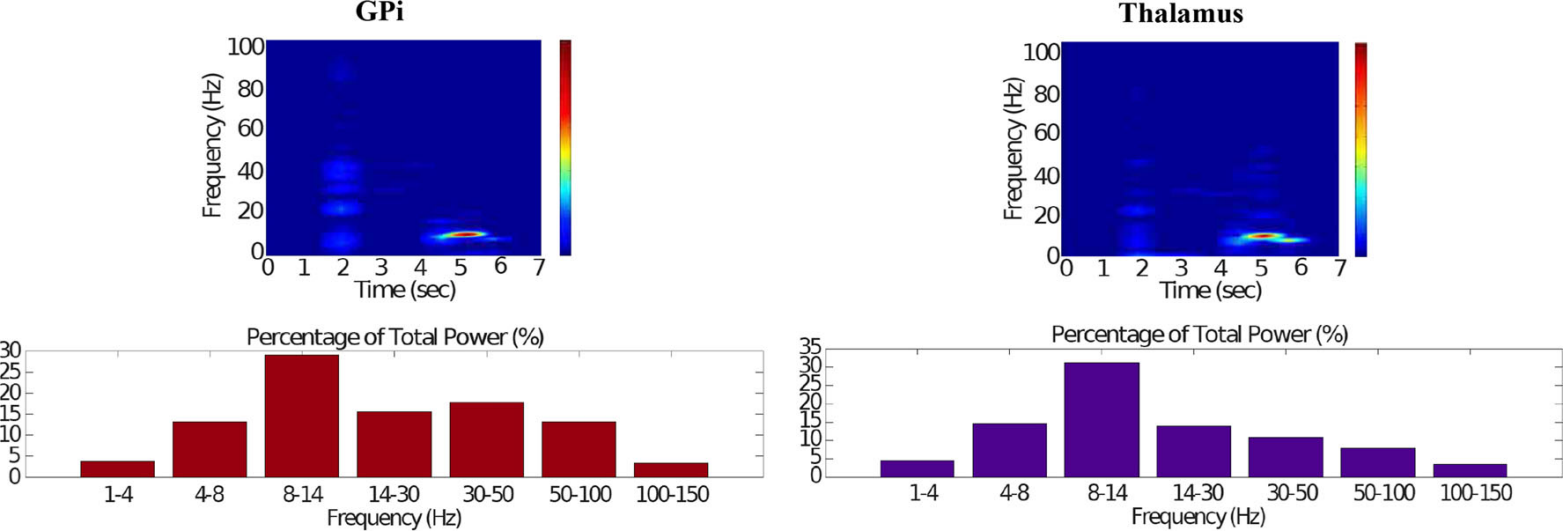
Healthy behaviour: adequate response to external stimulus with a high enough dopamine level to make the BG direct pathway dominant. Spectrogram and power histogram analysis results of dominant frequencies for the firing activity of the GPi and the thalamus. The GPi is adequately inhibited at alpha-frequency band (8–13 Hz) and the cortical information is loaded at the thalamus

We apply an external stimulus that lasts 2 s into the NMDA and GABA-receptor-type neurons of the posterior cortex (Figs. 12, 13, 14). This stimulation drives the excitatory neurons to an active persistent state. The activity of these neurons codes the external stimulus. We note that the activation of the BG direct pathway is associated with an over-activity of the $D_1$-MSNs and a reduced activity of the $D_2$-MSNs in the striatum, as Figs. 13 and 15 reflect. This is due to both the increased level of phasic DA and an upscaled efficacy of cortico-striatal synapses to $D_1$-MSNs, along with downscaled strength of cortico-striatal synapses to $D_2$-MSNs. At the same time, the $D_1$-MSNs reduce the inhibitory outflow from parts of the GPi (Figs. 13 and 16). Moreover, the thalamic disinhibition occurs as a result of the cascading dynamics within the BG network.

It is relevant to highlight that the STN-GPe subnetwork oscillates predominantly at gamma-frequency band (30–150 Hz), as Fig. 15 shows. On the one hand, the STN network is driven by the cortical excitation. On the other hand, the GPe is disinhibited through the indirect pathway. The overall effect over the GPi network is the inhibition by both the GPe and the $D_1$-MSN populations. This creates a window for the thalamic network to fire in response to a sensory stimulation. We can state the hypothesis that the inhibition is a control mechanism which leads to a rapid response in the thalamus, and is crucial for encoding and maintaining new information as it occurs. As a result of the circuitry considered, the striatal decision is mapped onto the thalamic population via the representation of the output layer: the GPi. The balance between the direct and indirect pathways ensures the execution of a given order.

In brief, the BG promotes action under the presence of new information by disinhibiting the associated target structures. As the transient phasic DA level decays, a transition from the ‘awake state’ to the ‘silent (quiescent) state’ occurs in the thalamus. Moreover, the power histograms in Figs. 15 and 16 prove that inter- and intra-network synchronisation is low, especially compared with the PD cases shown in “Action selection pathway enabled compatible with Parkinson’s disease” and “Default network mode compatible with Parkinson’s disease” sections. This is qualitatively compatible with experimental results in non-PD and PD cases (Obeso et al. 2008).

### Action selection pathway disabled

**Fig. 17:**
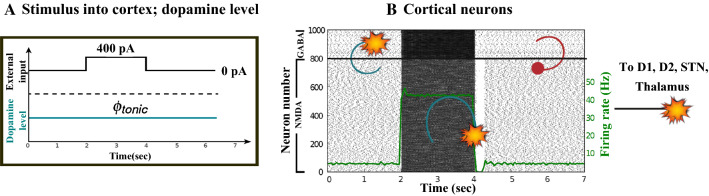
Healthy behaviour: adequate response to external stimulus with a low dopamine level ($\phi _{tonic}=0.5, \phi _{phasic}=0$) to make the BG indirect pathway dominant. **A** External stimulus applied to the cortical neural populations (NMDA and GABA-receptor populations) and variation of dopamine level $\phi (t)$. **B** Raster plot of the spiking activity of the simulated cortical neurons when the action selection pathway is disabled by a low phasic dopamine level ($\phi _{phasic}=0$) while an external stimulus is applied into the cortex. The NMDA-cortical neurons’ excitatory response is applied to the $D_1$-MSNs, the $D_2$-MSNs, the STN and the thalamus, and the result is given in Fig. 18. Each black dot corresponds to a spike of the corresponding neuron at a given time. The green function on the raster plot shows the average firing rate of the NMDA-cortical neurons for the 20 simulations produced. The raster plot is for one of these simulations. The sparks indicate excitatory connections, whereas the circles represent inhibitory connections

**Fig. 18:**
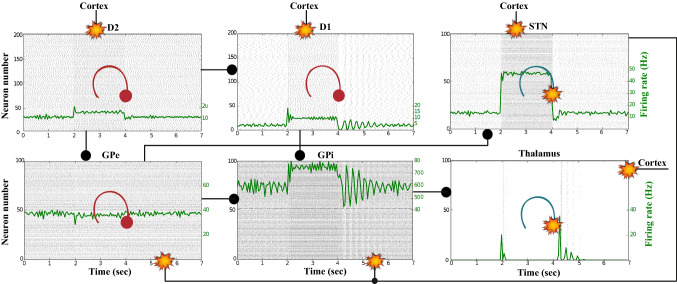
Healthy behaviour: adequate response to external stimulus with a low dopamine level ($\phi _{tonic}=0.5, \phi _{phasic}=0$) to make the BG indirect pathway dominant. Raster plots of the spiking activity of the relevant simulated populations of neurons when the action selection (direct) pathway is disabled by a low phasic dopamine level ($\phi _{phasic}=0$) while an external stimulus is applied into the cortex. The associated dopamine level, the external stimulus and the NMDA-cortical neurons’ response applied to the $D_1$-MSNs, the $D_2$-MSNs, the STN and the thalamus are given in Fig. 17. Each black dot corresponds to a spike of the corresponding neuron at a given time. For each population, the green functions on the raster plots show the average firing rate for the 20 simulations produced. The raster plots are for one of these simulations. The sparks indicate excitatory connections, whereas the circles represent inhibitory connections. The over-activity of the striatal $D_2$-MSNs suppresses the activity of the neurons of the GPe, which in turn are incapable of reducing the activity of the STN. The STN neurons start to send more excitatory inputs to the GPi to prevent the sensory input from reaching the thalamus

**Fig. 19:**
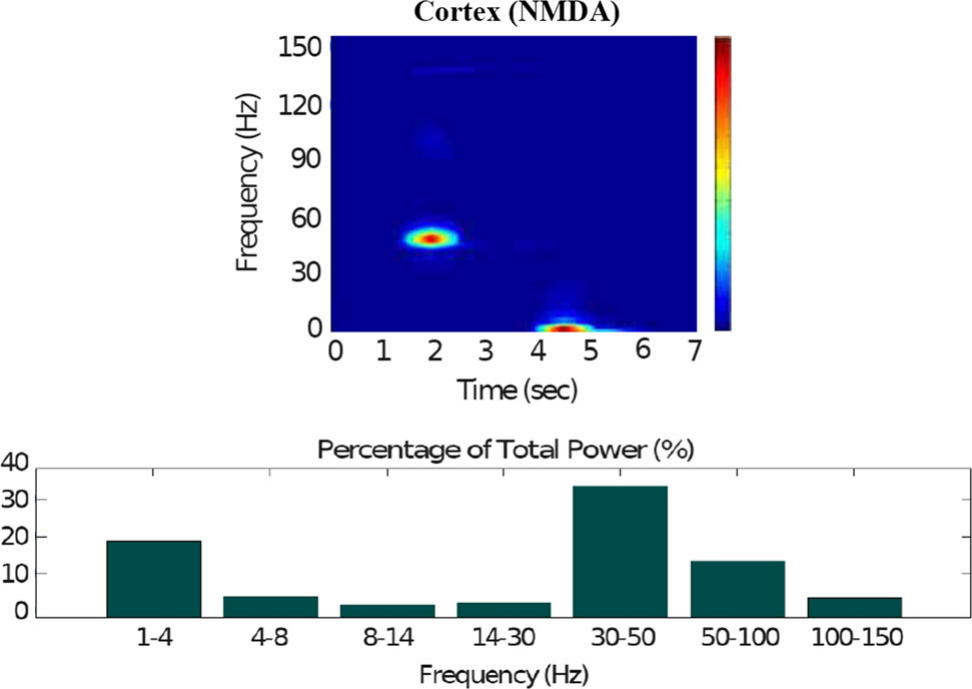
Healthy behaviour: adequate response to external stimulus with a low dopamine level to make the BG indirect pathway dominant. Spectrogram and power histogram analysis results of dominant frequencies for the firing activity of the NMDA-cortical neural population

**Fig. 20:**
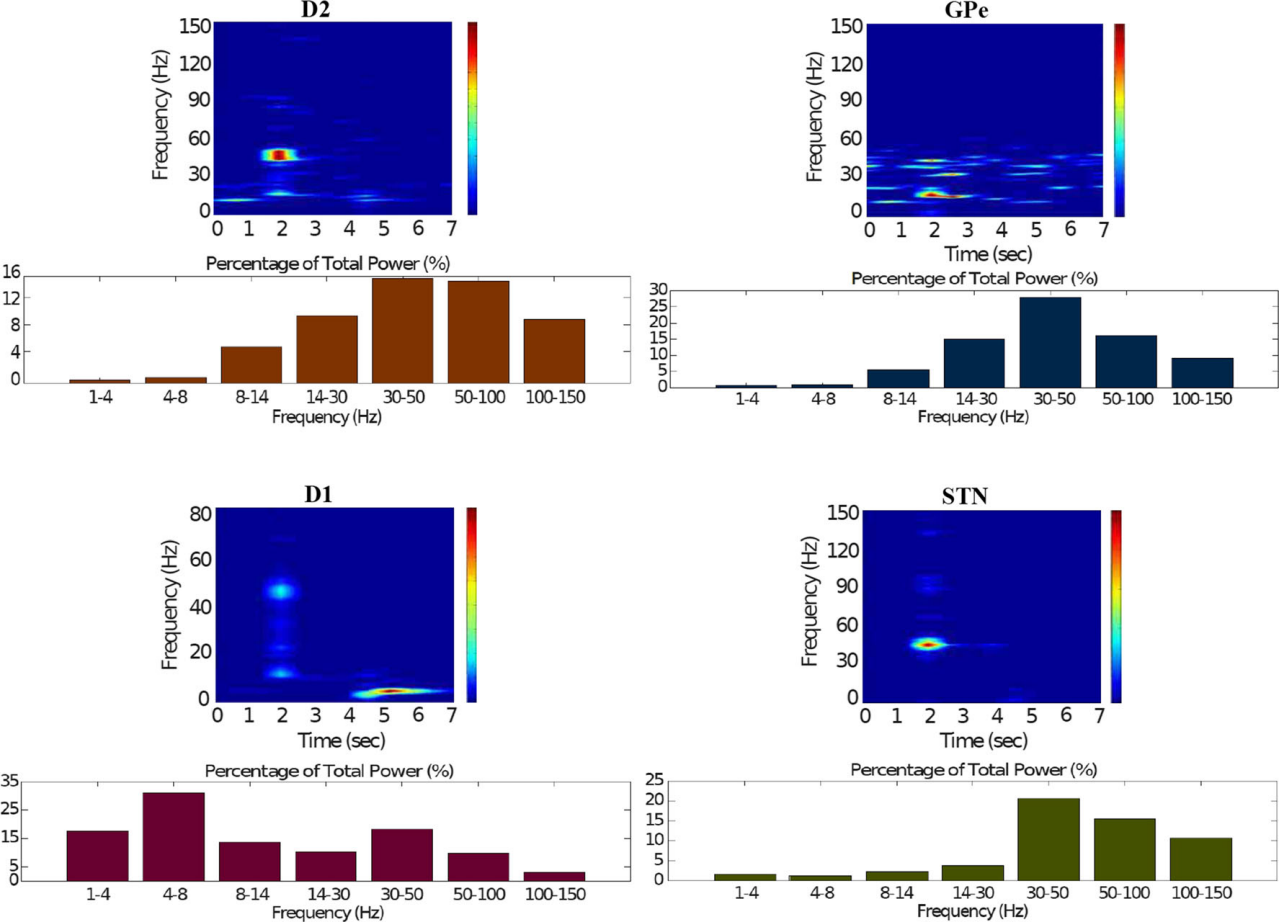
Healthy behaviour: adequate response to external stimulus with a low dopamine level to make the BG indirect pathway dominant. Spectrogram and power histogram analysis results of dominant frequencies for the firing activity of the $D_2$-MSNs, the GPe, the $D_1$-MSNs and the STN

Now we reproduce the case when the cortical neurons (Figs. 17, 18, 19) relay excitatory inputs to the $D_1$-MSNs and the $D_2$-MSNs, and they are incapable of causing enough stimulation in the $D_1$-MSNs to disinhibit the thalamic network through the direct pathway. This is due to the fact that the influence of the cortical neurons in the $D_1$-MSNs is scaled down by the low phasic DA level ($\phi _{phasic}=0$). Figures 18 and 20 show how the excitatory drive of the STN and the inhibitiory influence of the $D_2$-MSNs to the GPe network balance out each other, since the activity in the GPe population slightly changes during the input (2–4 s) and remains in the lower ranges of the gamma-frequency band (30–150 Hz).

**Fig. 21:**
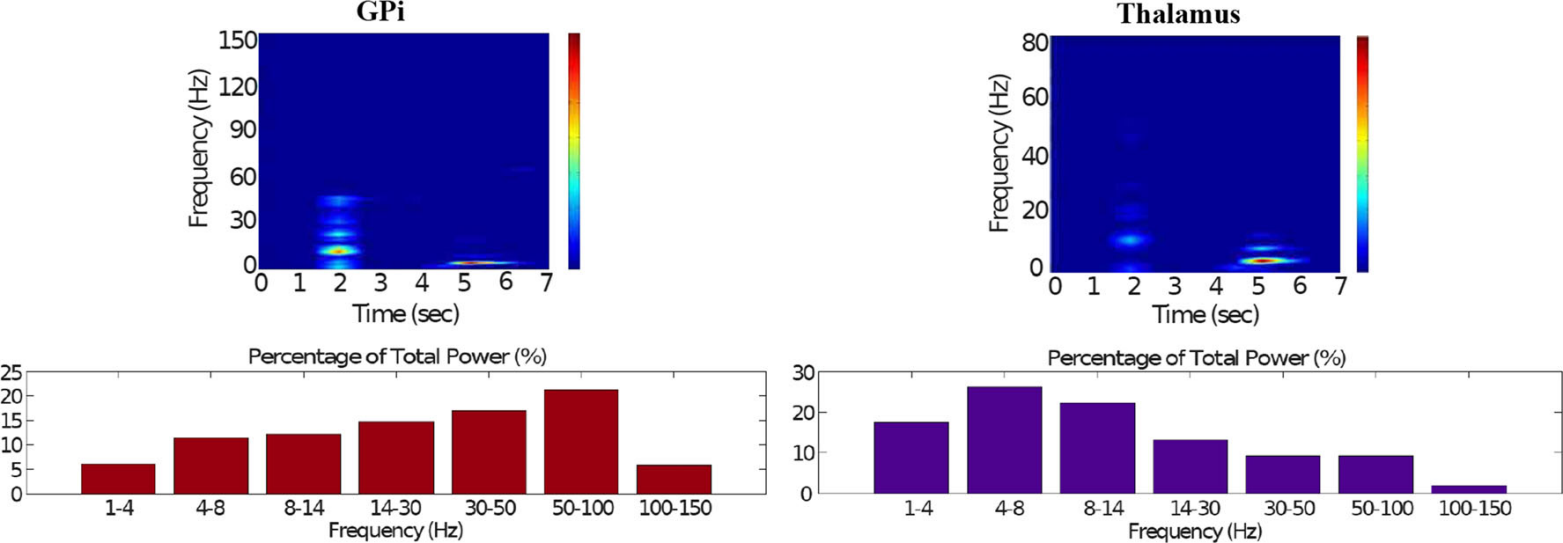
Healthy behaviour: adequate response to external stimulus with a low dopamine level to make the BG indirect pathway dominant. Spectrogram and power histogram analysis results of dominant frequencies for the firing activity of the GPi and the thalamus

In contrast to the case of “Action selection pathway enabled” section, where the action selection (direct) pathway is enabled, here, the STN excitation overrides the striatal inhibition over the GPi and provides an early stimulation through the hyperdirect pathway ($\text {Cortex}\Longrightarrow \text {STN}\Longrightarrow \text {GPi}\Longrightarrow \text {Thalamus}$). Within the cortical stimulation interval, the GPi network increases its activity up to a high gamma-frequency range (50–100 Hz), as Fig. 21 shows. As a result, the thalamus is only able to generate a brief response that fades, immediately after the GPi inhibition is produced.

Our model appropriately ‘locks the gates’ through dopaminergic modulation, even in the presence of a high-frequency cortical stimulation. After the stimulation interval, some thalamic neurons generate rebound bursts for a short time interval as they are disinhibited. As imaging studies have revealed, when the action selection pathway of the BG is disabled, a reduced thalamic activity and the onset of theta-frequency band activity (4–8 Hz) are produced. The power histogram results indicate the dominance of theta-frequency band oscillations in the $D_1$-MSNs and the thalamus (Figs. 20, 21), which drives the network to a regime such that a persistent active state in the network is prevented from happening, and an incoming signal can only produce a transient response.

### Action selection pathway enabled compatible with Parkinson’s disease

**Fig. 22:**
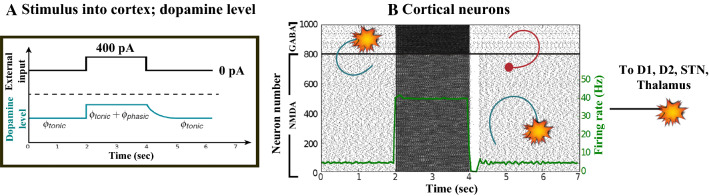
Activity compatible with PD: inadequate response to external stimulus when the BG direct pathway is dominant with $\phi _{tonic}=0.5$ and $\phi _{phasic}=0.4$. **A** External stimulus applied to the cortical neural populations (NMDA and GABA-receptor populations) and dopamine level (variation of $\phi (t)$). **B** Raster plot of the spiking activity of the simulated cortical neurons considered in our model when the BG action selection (direct) pathway is enabled but with a non-healthy dopamine level corresponding to a state compatible with PD. The NMDA-cortex population’s excitatory response is applied to the $D_1$-MSNs, the $D_2$-MSNs, the STN and the thalamus, and the result is given in Fig. 23. Each black dot corresponds to a spike of the corresponding neuron at a given time. The green function on the raster plot shows the average firing rate of the NMDA-cortical neurons for the 20 simulations produced. The raster plot is for one of these simulations. The sparks indicate excitatory connections, whereas the circles represent inhibitory connections

For the first PD-related network state, the evaluation set-up is the same as the healthy case presented in “Action selection pathway enabled” section. However, for the pathological case, the phasic DA level is attenuated ($\phi _{phasic}=0.4$) (Fig. 22). This causes an inadequate excitation of the $D_1$-MSN network, which remains incapable of providing inhibition over the GPi for the thalamic network to respond appropriately to an incoming input.

**Fig. 23:**
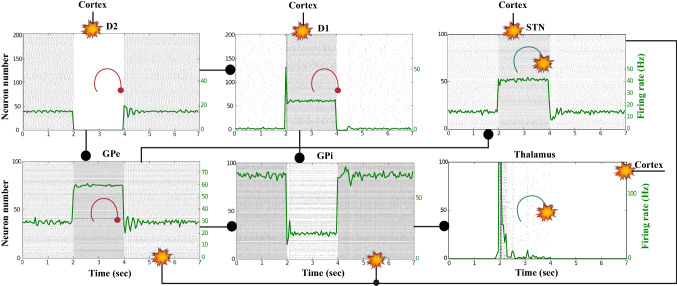
Activity compatible with PD: inadequate response to external stimulus when the BG direct pathway is dominant with $\phi _{tonic}=0.5$ and $\phi _{phasic}=0.4$. Raster plots of the spiking activity of the relevant simulated neural populations when the action selection pathway is enabled but with a non-healthy dopamine level corresponding to a state compatible with PD. The associated dopamine level ($\phi (t)$), the external stimulus and the NMDA-cortical neurons’ response applied to the $D_1$-MSNs, the $D_2$-MSNs, the STN and the thalamus are given in Fig. 22. Each black dot corresponds to a spike of the corresponding neuron at a given time. For each population, the green functions on the raster plots show the average firing rate for the 20 simulations produced. The raster plots are for one of these simulations. The sparks indicate excitatory connections, whereas the circles represent inhibitory connections

**Fig. 24:**
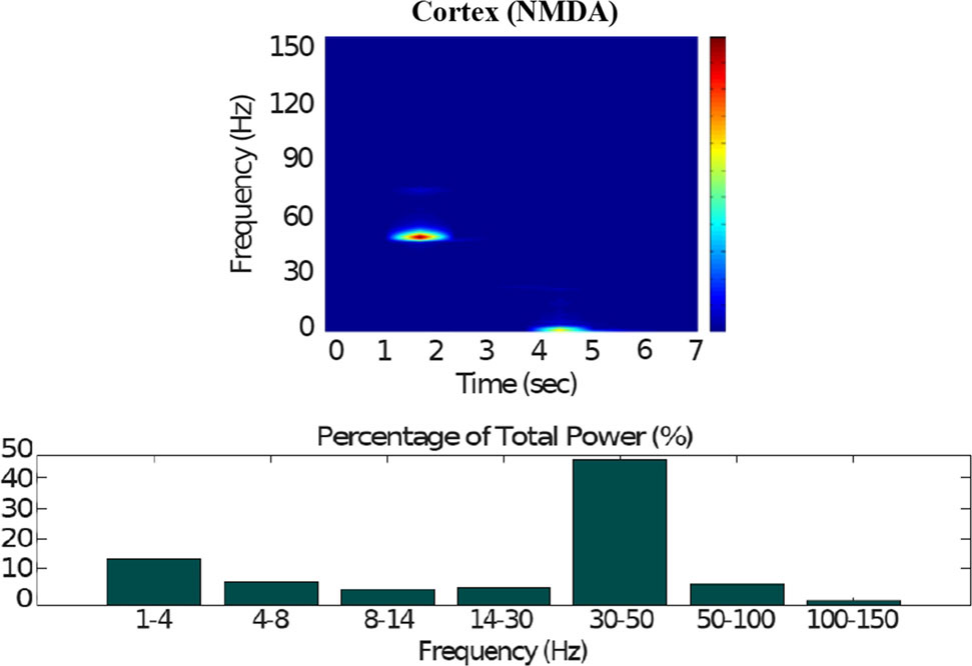
Activity compatible with PD: inadequate response to external stimulus when the BG direct pathway is dominant. Spectrogram and power histogram analysis results of dominant frequencies for the firing activity of the NMDA-cortical neural population

**Fig. 25:**
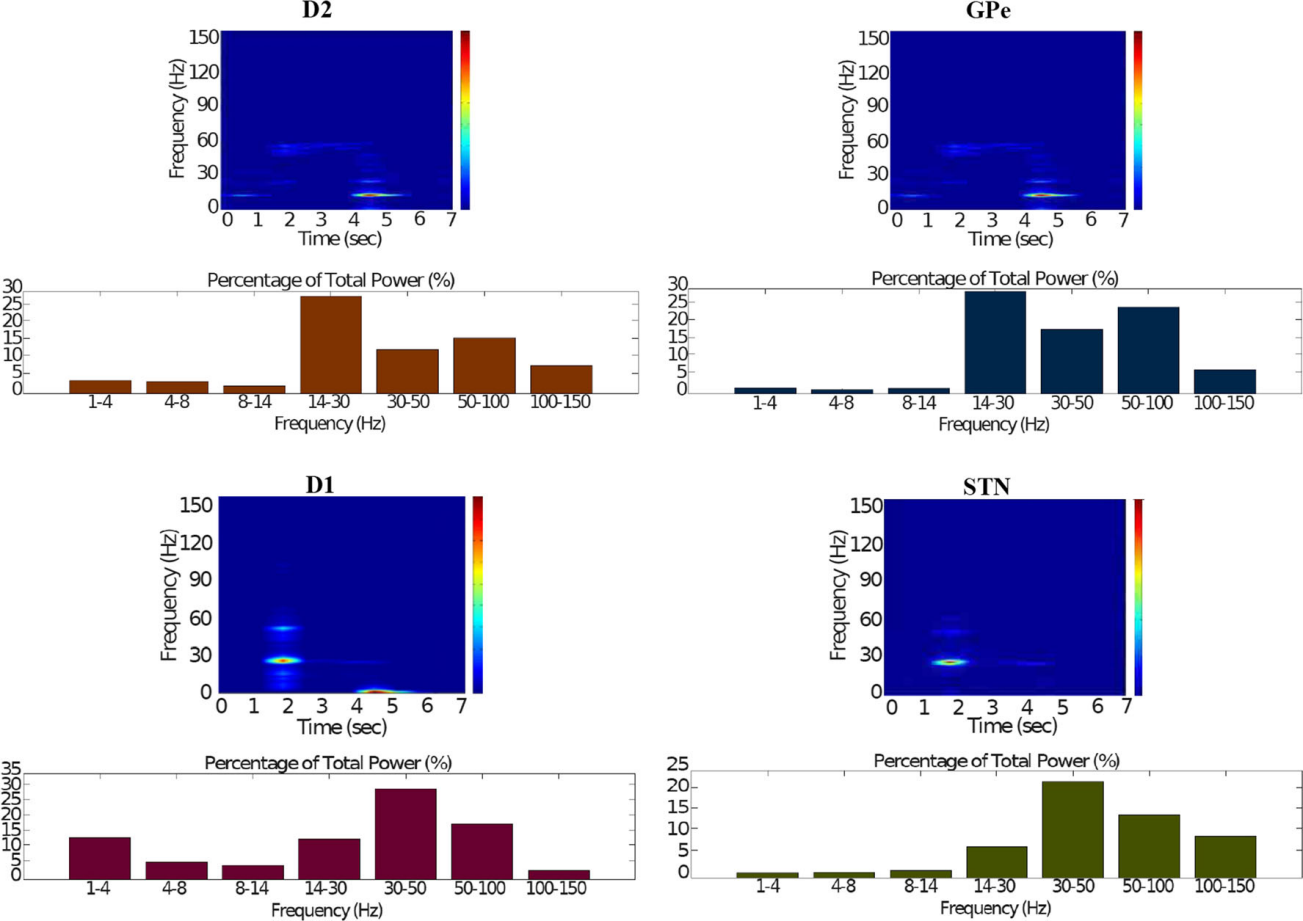
Activity compatible with PD: inadequate response to external stimulus when the BG direct pathway is dominant. Spectrogram and power histogram analysis results of dominant frequencies for the firing activity of the $D_2$-MSNs, the GPe, the $D_1$-MSNs and the STN. A low level of DA does not allow information to flow through the BG channels. There is a $D_2$-STN-GPe network synchronisation at beta-frequency band (13–30 Hz)

**Fig. 26:**
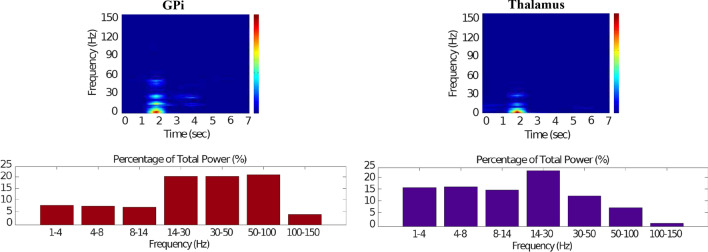
Activity compatible with PD: inadequate response to external stimulus when the BG direct pathway is dominant. Spectrogram and power histogram analysis results of dominant frequencies for the firing activity of the GPi and the thalamus. There is an increased activity of the GPi, which is not adequately inhibited by the $D_1$-MSNs. The thalamus (with activity at beta-frequency band) cannot respond to the cortical input appropriately

It can be concluded from Figs. 22, 23, 24, 25 and 26 that the GPi inhibition remains insufficient to successfully release the BG gate for the information transfer. The thalamic network can only generate a response in the theta-frequency band (4–8 Hz) within the stimulation interval. The GPe-STN subnetwork is tuned to couple at a lower frequency than in the healthy case presented in “Action selection pathway enabled” section, with a significant beta-frequency band activity in the GPe (13–30 Hz) as Fig. 25 shows. It can be also appreciated from the power histograms that the indirect pathway networks ($D_2$-MSNs, GPe, and STN) fire with an elevated synchronisation level, and the most prominent portion is at the beta-frequency band (13–30 Hz). We can then conclude that despite the action of the DA modulation, an imbalance in the inhibitory/excitatory firing may also contribute to the pathological activity in the network. This is compatible with reported dysfunctional PD-related activity ((Obeso et al. 2008) and references therein; further references are given in the Introduction).

### Default network mode compatible with Parkinson’s disease

**Fig. 27:**
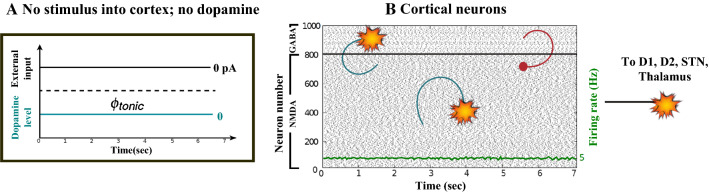
Activity compatible with resting tremors in PD. **A** No external stimulus is applied to the cortical neural populations (NMDA and GABA-receptor populations) and the level of dopamine is defined by $\phi (t)=\phi _{tonic}=0$ for all *t*. **B** Raster plot of the spiking activity of the simulated populations of the cortical neurons when no external stimulus is injected to the cortical network, the level of dopamine is the lowest one and an additional adjustment of the GPi-thalamus synaptic strengths is applied. The NMDA-cortical population’s excitatory response is applied to the $D_1$-MSNs, the $D_2$-MSNs, the STN and the thalamus, and the result is given in Fig. 28. Each black dot corresponds to a spike of the corresponding neuron at a given time. The green function on the raster plot shows the average firing rate of the NMDA-cortical neurons for the 20 simulations produced. The raster plot is for one of these simulations. The sparks indicate excitatory connections, whereas the circles represent inhibitory connections

**Fig. 28:**
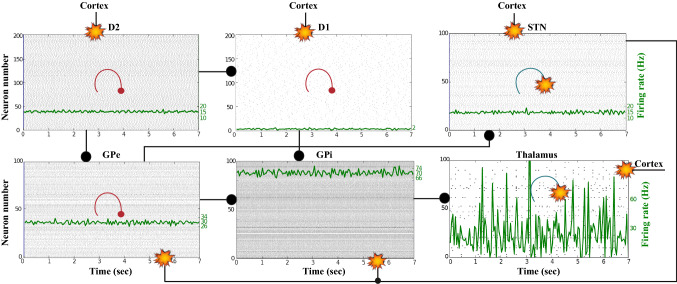
Activity compatible with resting tremors in PD. Raster plots of the spiking activity of the relevant simulated neural populations when no external stimulus is injected to the cortical network, the level of dopamine is defined by $\phi (t)=\phi _{tonic}=0$ for all *t*, and an additional adjustment of the GPi-thalamus synaptic strengths is applied. The associated dopamine level, the external stimulus applied to the cortex and the NMDA-cortical neurons’ response applied to the $D_1$-MSNs, the $D_2$-MSNs, the STN and the thalamus are given in Fig. 27. Each black dot corresponds to a spike of the corresponding neuron at a given time. For each population, the green functions on the raster plots show the average firing rate for the 20 simulations produced. The raster plots are for one of these simulations. The sparks indicate excitatory connections, whereas the circles are inhibitory connections

**Fig. 29:**
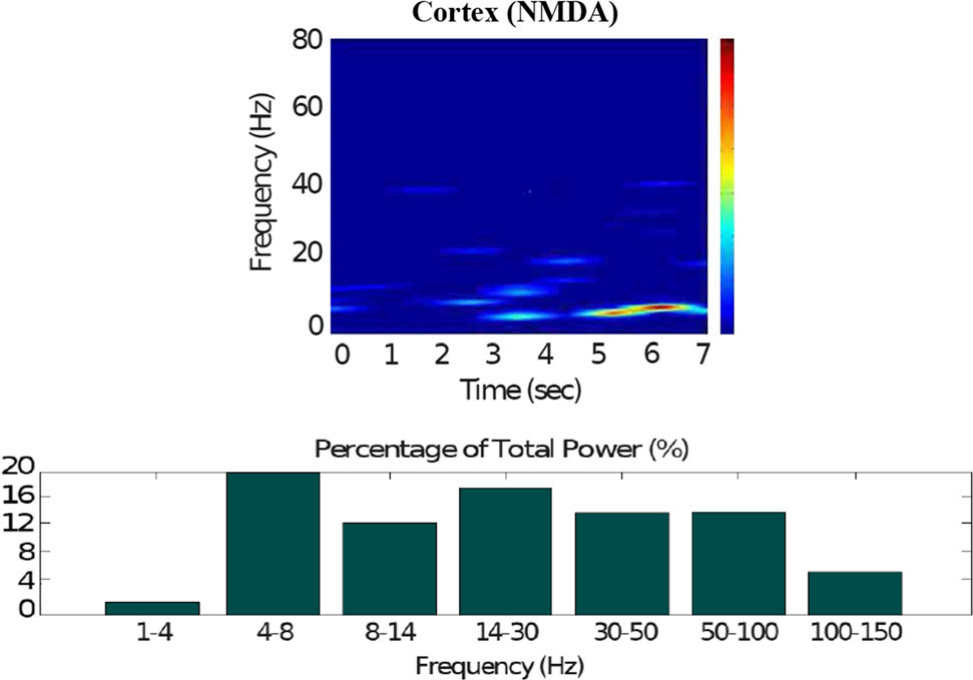
Activity compatible with resting tremors in PD. Spectrogram and power histogram analysis results of dominant frequencies for the firing activity of the NMDA-cortical neural population

**Fig. 30:**
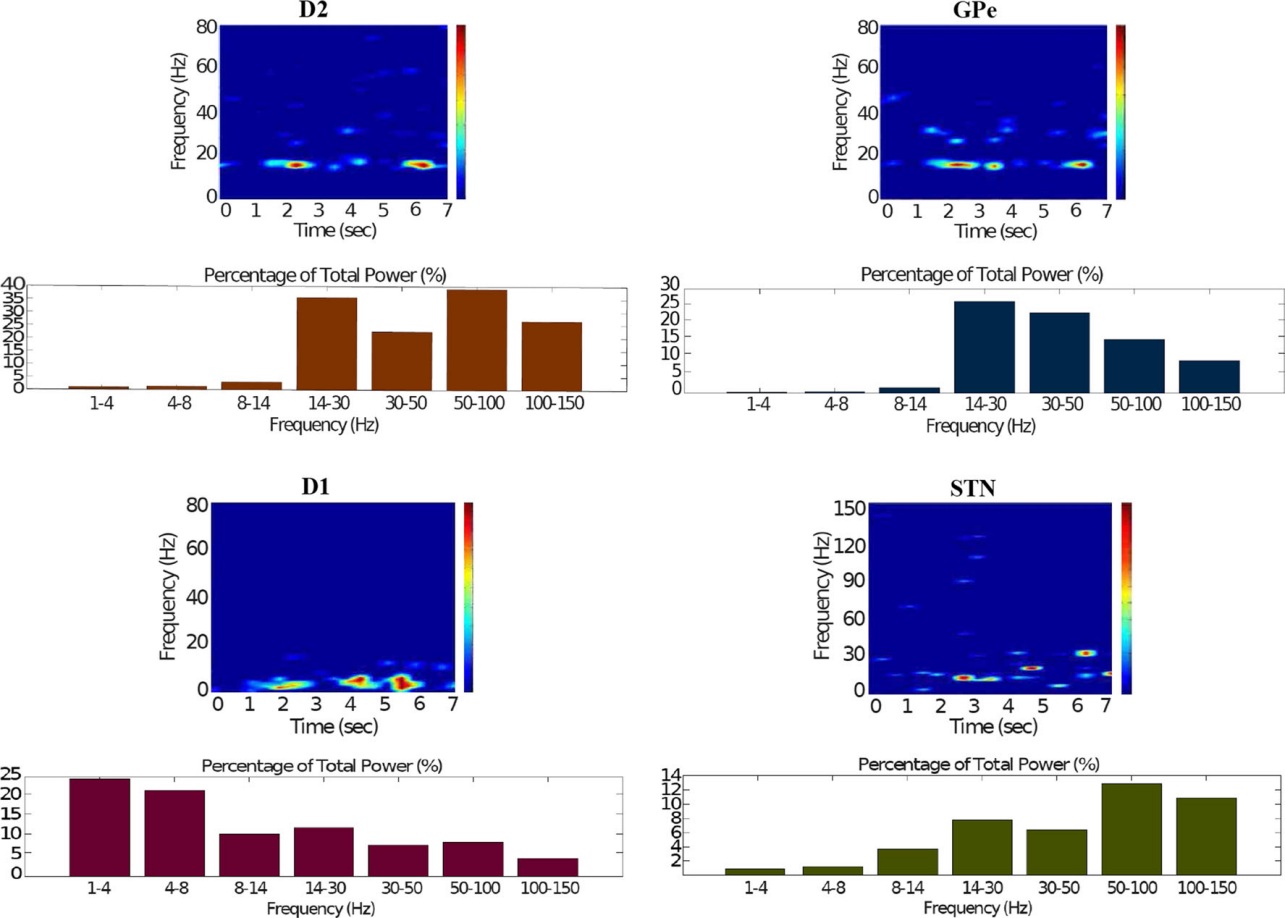
Activity compatible with resting tremors in PD. Spectrogram and power histogram analysis results of dominant frequencies for the firing activity of the $D_2$-MSNs, the GPe, the $D_1$-MSNs and the STN. There is a significant synchronisation of the $D_2$-STN-GPe network at beta-frequency band (13–30 Hz)

**Fig. 31:**
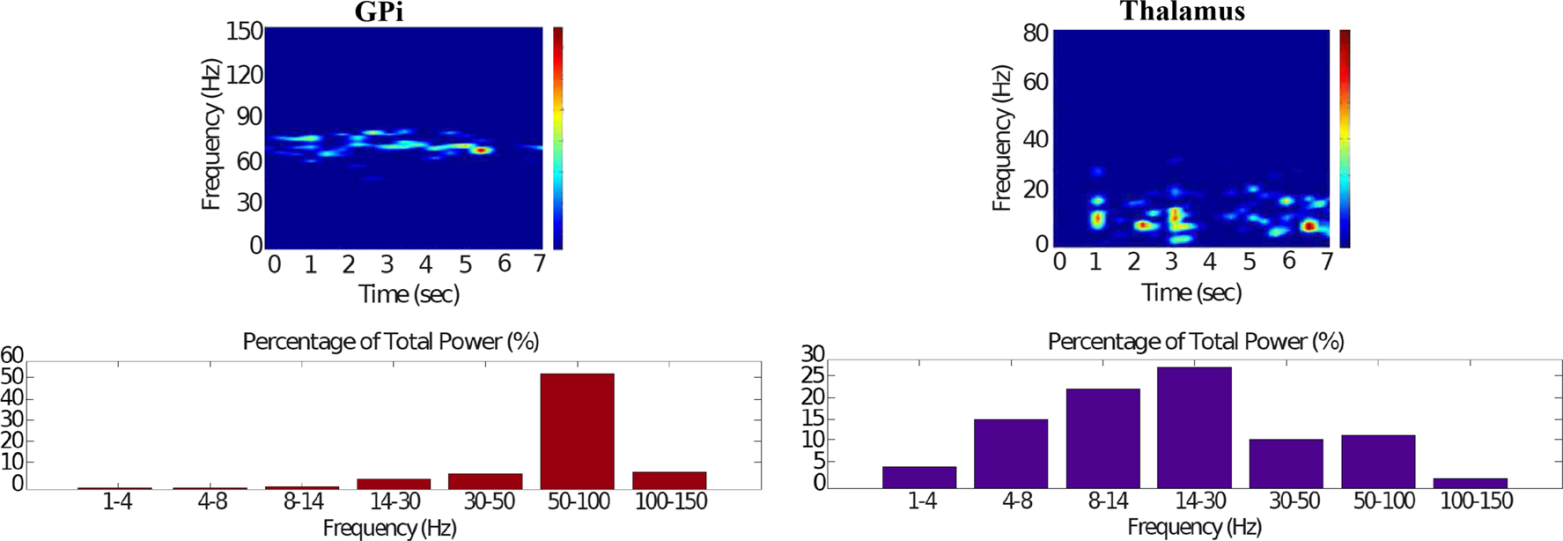
Activity compatible with resting tremors in PD. Spectrogram and power histogram analysis results of dominant frequencies for the firing activity of the GPi and the thalamus. The GPi presents an increased firing frequency and is not adequately inhibited by the $D_1$-MSNs. The thalamus (at beta-frequency band) reproduces tremor-like firing

Finally, we reproduce a Parkinsonian-like case when the BG network is ‘at rest’. The results are shown in Figs. 27, 28, 29, 30 and 31.

In this case, a low level of DA causes an excessive synchronisation in the BG network, particularly, in the beta-frequency range. However, the onset of tremor activity in the thalamus is triggered by the additional adjustment of the GPi-thalamus synaptic strengths. While maintaining the GPi-thalamus synaptic strengths fixed (no STDP update is applied), we reduce their values in comparison to the non-PD scenarios. Either the reduction of the tonic DA level or the reduction of the GPi-thalamus synaptic strengths alone was not sufficient to cause thalamic tremors, but both factors together may be considered as the cause of such a deficiency in the thalamic network. The need for an additional variation of the connections between the GPi and the thalamus reflects the well-known key role of the GPi on the activity of the thalamus in PD.

Figures 28 and 30, show that the STN, the $D_2$-MSNs and the GPe fire within the beta-frequency range (13–30 Hz) with a significant level of synchronisation. A low tonic DA level does not allow the network to communicate properly and efficiently due to an over-synchronisation of the network. The frequency analysis is even clearer: there is a significant increase in the beta-band power for the thalamus and all the BG nuclei, with the exception of the GPi (Figs. 30, 31). However, only considering a low DA level (as it was considered in case 1) of “Default network mode” section) was not enough to reproduce tremor-like activity in the thalamus. To achieve such a result, as it was explained above, we also reduced the synaptic strengths of the connections between the GPi and the thalamus. Under these conditions, the thalamic neurons generate three or more consecutive spikes as rebound bursts.

## Conclusions

We have proposed a novel dynamical model for the BG-thalamo-cortical network which reproduces the fine-tuning of the oscillatory activity of the BG with changing DA levels. Our model well demonstrates how different frequency-band oscillations can emerge due to the BG activity based on dopaminergic regulation. The framework proposed here links the switching behaviour of the BG to the regulation of the direct and indirect pathways depending on DA levels. The model has been validated through simulations for two main groups of scenarios: 1) healthy states, and 2) pathological states compatible with PD. The PD-related cases include attenuation of the DA level in the striatal circuitry. In the non-pathological or healthy cases, our model successfully mimics the experimentally-observed oscillations in the BG for three different scenarios in a qualitative way. It has been shown that the DA adjustment was able to act as a ‘gate holder’ in the model. In our simulations, the cortical information transmission is ensured by disinhibiting the thalamic network via an adequate DA release, or is blocked by the BG by avoiding thalamic excitation, as a consequence of an inadequate DA level during the sensory stimulus injection.

Our model has also shown how a reduced level of DA can contribute to the emergence of beta-frequency band oscillations within the BG-thalamic network, which is a signature of neural dynamic pathology in the BG in PD. Although our model is unlikely to capture the full complexity of PD, our limited, yet informative results, have shown that the dysfunction of DA cells in PD scenarios may make various biomarkers appear at different dynamical aspects of neural activity. Therefore, PD-related altered oscillatory activity might be a useful signature for defining a precondition in developing PD. PD is a result of a dysfunction in BG structures that can alter the capacity of selection and suppression of competing responses of the BG. Our model also displays similar difficulties both in suppressing thalamic activity—even when there is no external input–, and in appropriately transmitting the external input when the direct pathway is enabled by an adequate DA level. We also found that an inadequate DA level alone is not able to cause thalamic tremors. However, when a DA depletion is combined with a variation of GPi-thalamic synaptic strengths, a tremor-like activity emerges within the thalamic network. Nevertheless, it is inconclusive to directly link tremor activity to these two factors or to the limitations of the model. This issue needs further investigation.

The main advantage of our model is that it can qualitatively mimic experimentally-verified dynamics at both single-neuron and population levels. Immediate extensions and improvements of our model are as follows. First, the consideration of the evolution of the network to a self-sustained network. That is, a network that can show spontaneous and collective activity patterns without any external stimuli. Within this context, a self-sustained network may provide more information on the action selection of the BG and cognitive and motor-related tasks. Second, the BG model can be integrated into a working memory model by completing the loop with the addition of the thalamus-to-cortex circuit. Recently, we have proposed a multi-level hybrid automaton model for the switching mechanism of the BG and the thalamus which includes the thalamus-to-prefrontal cortex subnetwork and is linked to some working memory processes (Navarro-López et al. 2016; Çelikok et al. 2016). By merging these two strands of work, we may analyse cognitive and motor impairments together and associate them with pathological PD network states.

## Electronic supplementary material

Below is the link to the electronic supplementary material.

Supplementary material 1 (pdf 344 KB)
