# Supplementary material for: A dynamical model for the basal ganglia-thalamo-cortical oscillatory activity and its implications in Parkinson’s disease
Source: Cogn Neurodyn. 2020 Nov 25;15(4):693–720. doi: 10.1007/s11571-020-09653-y (PMC8286922; doi:10.1007/s11571-020-09653-y)
Supplement: Supplementary file 1 — Supplementary material 1 (pdf 344 KB) [file 11571_2020_9653_MOESM1_ESM.pdf]

Cognitive Neurodynamics manuscript No.
(will be inserted by the editor)

Electronic supplementary material for: A dynamical model for the basal ganglia-thalamo-cortical oscillatory activity and its implications in Parkinson's disease

Eva M. Navarro-López · Utku Çelikok · Neslihan S. Şengör

October 2020

Abstract This document provides the equations and the parameters needed to obtain the results presented in our paper. We first describe the models of the single neurons for all the relevant neural populations considered. Finally, we explain how the different populations of neurons are connected. For this, the evolution of the synaptic strengths over time is described.

1 Modelling Single Neurons

The neural populations considered in our model correspond to units of the posterior cortex, the striatum, the internal and the external segments of the globus pallidus (GPi and GPe), the subthalamic nucleus (STN) and the thalamus.

1.1 Model of the Posterior Cortex

Our model of the posterior cortex consists of two neural populations: excitatory neurons (glutamatergic with NMDA receptors) of the regular-spiking type, and inhibitory neurons (GABAergic) of the fast-spiking type. In the following sections, we will describe the dynamics for a single neuron of these two populations, and the model for the connections and synaptic plasticity between neurons.

E.M. Navarro-López (Corresponding author)
School of Mathematics and Computer Science University of Wolverhampton,
Alan Turing Building, Wulfruna Street, Wolverhampton WV1 1LY, United Kingdom
Tel.: +44-161-226-5461
E-mail: E.NavarroLopez@wlv.ac.uk.
ORCID ID: 0000-0001-8762-6943

U. Çelikok
Biomedical Engineering Department, Boğaziçi University, 34342, Beşiktaş, Istanbul, Turkey

N.S. Şengör
Department of Electronics and Telecommunication, Istanbul Technical University, Maslak, 34469, Sariyer, Istanbul, Turkey
ORCID ID: 0000-0001-6278-2392

1.1.1 Dynamical equations for the cortical neurons

For the cortical excitatory neurons, inspired by [3], the dynamics for the membrane potential ($v_{NMDA_{pCtx}}$) and the recovery current ($u_{NMDA_{pCtx}}$) for pyramidal neurons with NMDA-type receptors are described by the following equations. In the dynamical system below, $v_{NMDA_{pCtx}}$ and $u_{NMDA_{pCtx}}$ are vectors that contain the membrane potential and the recovery currents for all the neurons within the population of excitatory NMDA-type neurons within the posterior cortex:

$$
\begin{aligned}
C\frac{dv_{NMDA_{pCtx}}(t)}{dt} &= k\big[v_{NMDA_{pCtx}}(t) - v_r\big]\big[v_{NMDA_{pCtx}}(t) - v_t\big] - u_{NMDA_{pCtx}}(t)+ \\
&\quad + I_{NMDA_{pCtx}}(t), \\
\frac{du_{NMDA_{pCtx}}(t)}{dt} &= a\Big\{b\big[v_{NMDA_{pCtx}}(t) - v_r\big] - u_{NMDA_{pCtx}}(t)\Big\},
\end{aligned} \tag{1}
$$

where $v_{NMDA_{pCtx}} \in \mathbb{R}^{N_{NMDA_{pCtx}}}$, $u_{NMDA_{pCtx}} \in \mathbb{R}^{N_{NMDA_{pCtx}}}$, with $N_{NMDA_{pCtx}}$ the number of NMDA-type neurons within the posterior cortex. C is the membrane capacitance, $v_r \in \mathbb{R}^{N_{NMDA_{pCtx}}}$ the resting membrane potential for each neuron of the population and $v_t \in \mathbb{R}^{N_{NMDA_{pCtx}}}$ the instantaneous threshold potential for each neuron of the population. $I_{NMDA_{pCtx}}(t) \in \mathbb{R}^{N_{NMDA_{pCtx}}}$ is the total synaptic current flowing into every excitatory neuron at time t. The parameter a is the recovery time constant. Parameters k and b are derived from the single-neuron frequency-current ($f-I$) curve by considering the instantaneous firing-rate versus the net synaptic current. We also examined the results of the frequency analysis to reproduce, with the model, real-like electrophysiological behaviours of the firing activity within the neurons of the corresponding region of the brain.

We consider the following spike-generation and reset conditions of every element i of vectors $v_{NMDA_{pCtx}}$ and $u_{NMDA_{pCtx}}$ at $t_{peak_{NMDA_{pCtx}}}$, when the neuron fires:

$$
\text{for all } i,\ if \quad v_{NMDA_{pCtx}}(i) \geq v_{peak_{NMDA_{pCtx}}} \quad \text{then} \begin{cases} v_{NMDA_{pCtx}}(i) \leftarrow c \\ u_{NMDA_{pCtx}}(i) \leftarrow u_{NMDA_{pCtx}}(i) + d \end{cases} \tag{2}
$$

with $v_{peak_{NMDA_{pCtx}}}$ the spike cut-off value for the membrane potential, and c the voltage reset value – that is, the value of the membrane potential immediately after the neuron fires. The parameter d is tuned to achieve the desired rate of the spiking output.

The dynamical evolution of the membrane potential and the recovery current for GABAergic inhibitory neurons of the posterior cortex will follow similar equations to (1)-(2), but substituting the following variables and parameters $v_{NMDA_{pCtx}}$, $u_{NMDA_{pCtx}}$, $I_{NMDA_{pCtx}}$, and $v_{peak_{NMDA_{pCtx}}}$ by $v_{GABA_{pCtx}} \in \mathbb{R}^{N_{GABA_{pCtx}}}$, $u_{GABA_{pCtx}} \in \mathbb{R}^{N_{GABA_{pCtx}}}$, $I_{GABA_{pCtx}} \in \mathbb{R}^{N_{GABA_{pCtx}}}$ and $v_{peak_{GABA_{pCtx}}}$, respectively. The constant $N_{GABA_{pCtx}}$ is the number of GABAergic-type neurons within the posterior cortex. Here, the current $I_{GABA_{pCtx}}(t)$ models the total synaptic input current for inhibitory neurons in the posterior cortex.

The parameters C, k, a, v_r, v_t, b, c and d are different for inhibitory and excitatory neurons. Indeed, these parameters are different for each single neuron of our model, but we do not include a subscript in all of them for the sake of readability. The parameters used for NMDA-type and GABAergic neurons are given in Table 1.

1.1.2 Computation of synaptic currents and synaptic plasticity

The synaptic currents $I_{NMDA_{pCtx}}(t)$ and $I_{GABA_{pCtx}}(t)$ are given in equations (3) and (4), respectively:

$$I_{NMDA_{pCtx}}(t) = I_{Local}(t) - I_{pCtx_{GABA}}(t) + I_{ext}(t) + I_{back_{NMDA_{pCtx}}}(t), \tag{3}$$

$$I_{GABA_{pCtx}}(t) = I_{pCtx_{NMDA}}(t) + I_{ext}(t) + I_{back_{GABA_{pCtx}}}(t). \tag{4}$$

We will explain how we compute $I_{NMDA_{pCtx}}(t)$. Input $I_{GABA_{pCtx}}(t)$ is calculated in a similar way to $I_{NMDA_{pCtx}}(t)$.

In equations (3) and (4), $I_{ext}(t)$ represents the external incoming sensory information which will be loaded or ignored according to five different scenarios considered in Section 3 of the main article. The external incoming sensory stimulus is applied to both the inhibitory and the excitatory neurons of the posterior cortex. The external stimulus, represented by I_{ext}, is a temporary signal. Consequently, it has to be applied for a short-time interval. The input triggers the activation of the posterior cortex. The striatal neurons, the STN and the thalamus receive this cortical activity.

The input $I_{back_{NMDA_{pCtx}}}(t)$ in equation (3) represents the random background activity of the NMDA-receptor-type population in the posterior cortex. A background input is applied to all the populations of neurons, and is calculated separately for each of them. There are two main reasons to use a background input. First, the populations in the model do not only receive signals from the populations considered in the basal ganglia's circuit, but they also receive signals from other brain areas (long-range connections), and this activity is represented by the background input. Furthermore, the background input gives the possibility to reach the threshold needed for the activation of populations and to allow them to have a random firing activity in the absence of any other input. The background current is modelled as random values generated with a Poisson probability distribution for GABAergic inhibitory neurons. For excitatory NMDA neurons, the background current is modelled with random positive values generated from a standard uniform distribution. The background input current is applied during the total duration of the simulation.

Poisson probability distributions provide a good description for irregularities in neurons' spike times. This formulation generates the probability density of a single-spike train. With a Poisson probability distribution, each spike is generated independently from other spikes with an instantaneous firing rate, and these generated spikes are used as an input. Thus, the background inputs can be thought as the firing rate of a pre-synaptic population. In the simulations, we use the *poissrnd* command from the Statistics Toolbox of MATLAB to generate the Poisson probability distributions. This function returns the value of a number of spikes which will be used as the background for each time step and each neuron.

$I_{back_{NMDA_{pCtx}}}(t)$ is a vector of dimension $N_{NMDA_{pCtx}}$, and each element of them corresponds to the input of each neuron of the population. $I_{ext}(t)$ is a vector of dimension $N_{NMDA_{pCtx}}$ for equation (3), and a vector of dimension $N_{GABA_{pCtx}}$ for equation (4).

The currents $I_{Local}(t)$ and $I_{pCtx_{GABA}}(t)$ are also vectors of dimension $N_{NMDA_{pCtx}}$. The current $I_{pCtx_{GABA}}(t)$ models the contribution of inhibitory GABAergic neurons to NMDA-receptor-type neurons within the posterior cortex, and $I_{Local}(t)$ is the recurrent activity of excitatory NMDA-receptor-type neurons in the posterior cortex. Recurrent activity of a neural population is crucial, especially for robust maintenance of self-sustained persistent neural activity in the absence of external stimuli. These synaptic input currents are calculated with the following expressions, where we consider an axonal conduction delay:

$$\begin{aligned} I_{Local}(t) &= \sum_{t_n} r S_{NMDA_{pCtx}} \delta_V(t - (t_n + t_d)), \\ I_{pCtx_{GABA}}(t) &= \sum_{t_n^*} r S^*_{pCtx_{GABA}} \delta^*_V(t - (t^*_n + t^*_d)), \end{aligned} \tag{5}$$

where t_n and t_d are the spike time and the axonal conduction delay of a pre-synaptic NMDA-receptor-type neuron in the posterior cortex, respectively. The parameter r is a random number which is generated uniformly in the interval $[0, 1]$, $\delta_V(t - (t_n + t_d))$ is a vector of dimension $N_{NMDA_{pCtx}} \times 1$ with each element as a Dirac delta function $\delta(t - (t_n + t_d))$ that results in the incremental increase of I_{Local} at each time $t_n + t_d$.

On the other hand, t^*_n and t^*_d are the spike time and the axonal conduction delay of a pre-synaptic GABAergic neuron of the posterior cortex, respectively. Moreover, $\delta^*_V(t-(t^*_n+t^*_d))$ is a

vector of dimension $N_{GABA_{pCtx}} \times 1$ with each element as a Dirac delta function $\delta(t-(t_n^*+t_d^*))$ that results in the incremental increase of $I_{pCtx_{GABA}}$ at each time $t_n^* + t_d^*$.

The time-varying connections between the pre-synaptic neuron population and the post-synaptic neuron population are represented by the connection matrices:

$$S_{NMDA_{pCtx}} \in \mathbb{R}^{N_{NMDA_{pCtx}} \times N_{NMDA_{pCtx}}},$$
$$S^*_{pCtx_{GABA}} \in \mathbb{R}^{N_{NMDA_{pCtx}} \times N_{GABA_{pCtx}}}.$$

We note that, in our model, for the case of an NMDA-type excitatory neuron of the posterior cortex (post-synaptic neuron here), the pre-synaptic neuron that may fire can belong to two populations of neurons, namely: NMDA-type neurons of the posterior cortex and GABAergic inhibitory neurons of the posterior cortex.

Let us consider the computation of the input $I_{Local}(t)$ in equation (5) for NMDA-type neurons of the posterior cortex. In this case, each element (i, j) of the connection matrix $S_{NMDA_{pCtx}}$ is the synaptic strength between the ith pre-synaptic NMDA-receptor-type neuron that has fired at time t_n and the jth post-synaptic NMDA-receptor-type neuron. The elements $S_{NMDA_{pCtx}}(i, j)$, for all $(i, j) \in N_{NMDA_{pCtx}} \times N_{NMDA_{pCtx}}$ such that $i = j$, are considered 0 to indicate that it is not possible to have a connection between a neuron and itself. With an abuse of notation each $S_{NMDA_{pCtx}}(i, j)$ will be denoted by $s_{NMDA_{pCtx}}$ and be referred to as synaptic strength. We consider each synaptic strength $s_{NMDA_{pCtx}}$ as a constant parameter for most of the synapses. Time-varying synaptic plasticity is only considered for the connections within the cortical NMDA-type neurons, the connections between the cortical NMDA-type and GABAergic neurons, and finally, for the connections between the cortical NMDA-type neurons and the D_1-MSNs. The variation over time of these synaptic strengths is modelled by a spike-timing-dependent plasticity (STDP) model.

In our STDP model, for the update of the synaptic strength, we need to detect whether or not the pre-synaptic and the post-synaptic neurons are firing together. Let us consider t_n as the time a pre-synaptic neuron fires, and t_{peak} as the time a post-synaptic neuron fires. If $t_n = t_{peak}$ then the pre- and post-synaptic neurons fire together. Since, we consider axonal conduction delays, we need to add to t_n the time t_d, and $t_n + t_d$ will be the time at which the post-synaptic neuron can detect that the pre-synaptic neuron has fired, and this time will be denoted by $t_{nd} = t_n + t_d$.

Detecting $t_{nd} = t_{peak}$ in simulation, which is the same as detecting that $t_{nd} - t_{peak} = 0$, is difficult. To avoid this problem, we introduce a region around the zero of the expression $t_{nd} - t_{peak}$, whereby if the magnitude of $t_{nd} - t_{peak}$ is less than a small number, say $\Delta t > 0$, we can either have $t_{nd} = t_{peak}$ (firing together) or be in transition to the state of firing together. Defining this region makes it easier to detect when $t_{nd} - t_{peak}$ is close to zero. We will distinguish two transitions states to $t_{nd} - t_{peak} = 0$:

1) $t_{nd} - t_{peak} \leq 0$, or
2) $t_{nd} - t_{peak} > 0$.

To sum up, the idea behind our STDP model is the following one. Let us consider a presynaptic ("sending") neuron A and a postsynaptic ("receiving") neuron B. Let us define t_{peak_A} as the time where neuron A fires, t_{peak_B} as the time where neuron B fires, and t_{d_A} as the axonal conduction delay of neuron A, that is, the time that it takes for neuron B to detect that neuron A has fired. Let us also consider two constants $\omega_{t_{peak_B}} \in (0, 1]$, $J_{A,B} \in (0, 1]$:

- If A and B **are not close to firing together**, the synaptic strength between these two neurons $s_{A,B}$ is unchanged.
- If A and B **are close to firing together**, we consider two cases:
  1. If **neuron A fires before neuron B**, the synaptic strength $s_{A,B}$ is updated in the following manner:
  $$s_{A,B} \leftarrow s_{A,B} + \omega_{t_{peak_B}} J_{A,B}.$$
  2. If **neuron B fires before neuron A**:
  $$s_{A,B} \leftarrow s_{A,B} - \omega_{t_{peak_B}} J_{A,B}.$$

This can be better understood by examining Fig. 1.

Let us apply the STDP rule for the connections between cortical NMDA neurons. For the NMDA-neuron case, the synaptic strength $s_{NMDA_{pCtx}}$ is obtained in the following manner for every time a pre-synaptic neuron (an NMDA-type neuron of the posterior cortex) fires at time t_n and the synaptic strength is modified with delay t_d:

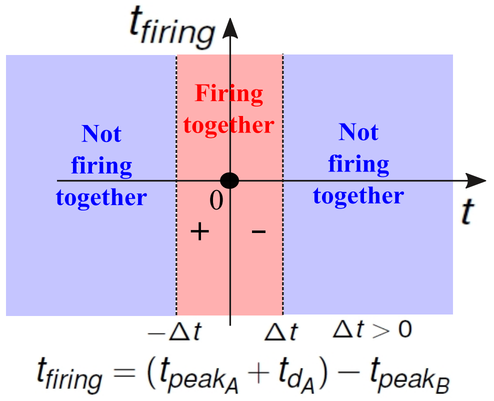

Fig. 1: Time-firing conditions for establishing the spike-timing-dependent plasticity (STDP) rules to update the synaptic strength between a presynaptic ("sending") neuron A and a postsynaptic ("receiving") neuron B. Remark: in the equations of our model, the time when a pre-synaptic neuron fires is denoted by t_n.

– For every n, if $\left| t_{nd} - t_{peak_{NMDA_{pCtx}}} \right| > \Delta t$ (**pre- and post-synaptic neurons do not fire together**):

$$s_{NMDA_{pCtx}} \leftarrow s_{NMDA_{pCtx}}. \tag{6}$$

– For every n, if $\left| t_{nd} - t_{peak_{NMDA_{pCtx}}} \right| \leq \Delta t$ and $t_{nd} \leq t_{peak_{NMDA_{pCtx}}}$ (**transition 1 to firing together**):

$$s_{NMDA_{pCtx}} \leftarrow s_{NMDA_{pCtx}} + J_{inc_{NMDA_{pCtx}}} \omega \delta(t - t_{nd}). \tag{7}$$

– For every n, if $\left| t_{nd} - t_{peak_{NMDA_{pCtx}}} \right| \leq \Delta t$ and $t_{nd} > t_{peak_{NMDA_{pCtx}}}$ (**transition 2 to firing together**):

$$s_{NMDA_{pCtx}} \leftarrow s_{NMDA_{pCtx}} - J_{inc_{NMDA_{pCtx}}} \omega \delta(t - t_{nd}), \tag{8}$$

where $t_{peak_{NMDA_{pCtx}}}$ is the time an $NMDA$-type excitatory neuron of the posterior cortex (now, post-synaptic neuron) fires. $J_{inc_{NMDA_{pCtx}}} \in (0, 1]$ is a parameter which is the same for each neuron within the same population and is different for different types of neurons. For the NMDA cortical neurons, we have $J_{inc_{NMDA_{pCtx}}} = 0.03$. The parameter ω, with a value between 0 and 1, depends on the dopamine level $\phi(t)$ (defined in Section 1.2.1). It is considered as $\omega = \phi(t)/100 + 0.002$, with t the time the pre-synaptic and the post-synaptic neurons fire simultaneously (or are very close to fire together, to be precise). Thus, the value of $s_{NMDA_{pCtx}}$ is changed with $J_{inc_{NMDA_{pCtx}}}$ and ω. In our evolving connections, the synaptic strength between two connected neurons is kept the same if their firing times do not coincide, but it is changed whenever their firing times coincide (or are close to coincide, to be precise). These parameters, which define the connection dynamics, are given in Table 10. More details are given in Section 2.

In an analogous way, we compute each element of the connection matrix $S^*_{pCtx_{GABA}}$. That is, the synaptic strengths $s^*_{pCtx_{GABA}}$ between the pre-synaptic GABAergic neurons of the posterior cortex and the post-synaptic NMDA-type neurons of the posterior cortex are calculated in the following way:

– For every n, if $\left| t^*_{nd} - t_{peak_{NMDA_{pCtx}}} \right| > \Delta t$:

$$s_{pCtx_{GABA}} \leftarrow s_{pCtx_{GABA}}. \tag{9}$$

– For every n, if $\left|t_{nd}^{*}-t_{peak_{NMDA_{pCtx}}}\right| \leq \Delta t$ and $t_{nd}^{*} \leq t_{peak_{NMDA_{pCtx}}}$:

$$s_{pCtx_{GABA}} \leftarrow s_{pCtx_{GABA}} + J^{*}_{inc_{NMDA_{pCtx}}} \omega^{*} \delta(t-t_{nd}^{*}). \tag{10}$$

– For every n, if $\left|t_{nd}^{*}-t_{peak_{NMDA_{pCtx}}}\right| \leq \Delta t$ and $t_{nd}^{*} > t_{peak_{NMDA_{pCtx}}}$:

$$s_{pCtx_{GABA}} \leftarrow s_{pCtx_{GABA}} - J^{*}_{inc_{NMDA_{pCtx}}} \omega^{*} \delta(t-t_{nd}^{*}), \tag{11}$$

where $t_{nd}^{*} = t_{n}^{*} + t_{d}^{*}$.

For the computation of $I_{GABA_{pCtx}}(t)$, in equation (4), the term $I_{pCtx_{NMDA}}(t)$ represents the contribution of $NMDA$-type excitatory neurons of the posterior cortex to GABAergic inhibitory neurons of the posterior cortex; $I_{back_{GABA_{pCtx}}}(t)$ is the background activity of GABAergic inhibitory neurons of the posterior cortex. $I_{back_{GABA_{pCtx}}}(t)$ is obtained from random values generated with a Poisson probability distribution.

The synaptic input current $I_{pCtx_{NMDA}}(t)$ appearing in equation (4) for the computation of $I_{GABA_{pCtx}}(t)$ is obtained in an analogous way as (5)–(8), substituting $pCtx_{GABA}$ by $pCtx_{NMDA}$ and considering appropriate dimensions of the involved vectors and matrices depending on the number of neurons of the population of GABAergic neurons and the population of pre-synaptic NMDA neurons.

The parameters used for all the connections between neural populations are given in Section 2.

Table 1: Parameters for the models of the neurons of the posterior cortex. Random values ($rand$) are added to v_r so that neurons of the same type do not fire at the same time

Parameter	Description	NMDA-receptor	GABA-receptor
N	Number of neurons	800	200
C	Capacitance	125pF	100pF
v_r	Reset potential	-74+3$rand$ mV	-68-3$rand$ mV
v_t	Instantaneous threshold potential	-54mV	-54mV
a	Recovery time constant	0.01	0.5
k	Tuned for appropriate firing rate	1	1.4
b	Tuned for appropriate firing rate	0.1	0.1
c	Parameter in the reset of the membrane potential	-75	-65
d	Parameter in the reset of the recovery current	25	10
v_{peak}	Spike threshold	30mV	30mV

1.2 Model of the Striatum

The proposed model of the striatum consists of two different populations: D_1-receptor type neurons and D_2-receptor type neurons. D_1 and D_2-receptor type neurons are medium spiny projection neurons (MSNs).

1.2.1 Model for D_1-MSNs

Inspired by [1–4], the dynamics for the membrane potential (v_{D_1}) and the recovery current (u_{D_1}) for striatal MSNs with D_1-type receptors are:

$$\begin{aligned} C\frac{dv_{D_1}(t)}{dt} &= k\big[v_{D_1}(t)-v_r\big]\big[v_{D_1}(t)-v_t\big] - u_{D_1}(t) + I_{D_1}(t) + 2\phi_1(t) g_{DA}\big[v_{D_1}(t)-E_{DA}\big], \\ \frac{du_{D_1}(t)}{dt} &= a\Big\{b\big[v_{D_1}(t)-v_r\big] - u_{D_1}(t)\Big\}, \end{aligned} \tag{12}$$

where $v_{D_1} \in \mathbb{R}^{N_{D_1}}$, $u_{D_1} \in \mathbb{R}^{N_{D_1}}$, with N_{D_1} the number of MSNs with D_1-type receptors within the striatum. C is the membrane capacitance, $v_r \in \mathbb{R}^{N_{D_1}}$ the resting membrane potential for each neuron of the population and $v_t \in \mathbb{R}^{N_{D_1}}$ the instantaneous threshold potential for each neuron of the population. $I_{D1}(t) \in \mathbb{R}^{N_{D_1}}$ is the total synaptic current flowing into every neuron at time t. The parameter a is the recovery time constant; k and b are derived from the single-neuron frequency-current $(f - I)$ curve by considering the instantaneous firing-rate versus the net synaptic current. $E_{DA} \in \mathbb{R}^{N_{D_1}}$ and $g_{DA} \in \mathbb{R}$ is the reversal potential for each neuron of the D_1-MSN population and the conductance of dopamine regulation, respectively. All the elements of vector E_{DA} are the same.

The function $\phi_1(t) \in \mathbb{R}$, with values in $[0, 1]$, models the proportion of active dopamine in the D_1-type receptors. Values of ϕ_1 close to 1 result in the over activation of D_1-type receptors by modulating both cortical input (see equation for $I^*_{pCtx_{NMDA_{D_1}}}(t)$ below) and the membrane potential dynamics.

For the D_2-type receptors, we use the function $\phi_2(t) \in \mathbb{R}$, with values in $[0, 1]$, which appears in the membrane potential dynamics for the MSNs with D_2-type receptors (equation (16)). The higher the value of ϕ_2 is, the more inhibition in D_2 receptors is produced. ϕ_1 and ϕ_2 can be interpreted as input control functions. In this work, they are time-varying to show different dominant states in the network. We will consider $\phi_1(t) = \phi_2(t) = \phi(t)$ for all t.

The value of $\phi(t)$ is determined by the tonic and the phasic levels of the dopamine. The tonic value (ϕ_{tonic}) determines the steady state of the dopamine concentration and is considered constant. We will consider $\phi(t) = \phi_{tonic}$, for all t, when there is no external stimulus in the cortex. When an external stimulus is applied to the cortex, we will also consider a time-varying phasic evolution of the dopamine, which will model the transient dopamine concentration that will decay with time after the cortical stimulus disappears. The distinction between tonic and phasic dopamine levels gives a more realistic approximation of how the level of dopamine varies into the basal ganglia.

All these features can be expressed mathematically in the following way. Let us consider two constants $\phi_{tonic}, \phi_{phasic} \in \mathbb{R}$, with $\phi_{tonic} \geq 0$ and $\phi_{phasic} \geq 0$ such that the sum of $\phi_{tonic} + \phi_{phasic} \in [0, 1]$, a parameter $\eta \in \mathbb{R}$, with $\eta \in (0.1)$, and $T = [t_1, t_2]$ the interval of time when an external stimulus is applied to the cortex, with $t_1, t_2 \in \mathbb{R}$ and $0 \leq t_1 < t_2$. Then we define $\phi(t)$ as a switching function with the following form:

$$\phi(t) = \begin{cases} \phi_{tonic} & \text{if } 0 \leq t < t_1, \\ \phi_{tonic} + \phi_{phasic} & \text{if } t \in [t_1, t_2], \\ \phi_{tonic} + \phi_{phasic} \left(e^{-\eta t}\right) & \text{if } t > t_2. \end{cases} \tag{13}$$

If no external stimulus is applied in the cortex, we consider $\phi(t) = \phi_{tonic}$ for all t.

For the simulation of the different scenarios described in Section 3 of the main paper, we will give the values for the different parameters of $\phi(t)$.

The spike-generation and reset conditions of every element i of vectors v_{D_1} and u_{D_1} at $t_{peak_{D_1}}$, when the D_1-MSN neuron fires, are:

$$\text{for all } i, \text{ if } \; v_{D_1}(i) \geq v_{peak_{D_1}} \; \text{ then} \begin{cases} v_{D_1}(i) \leftarrow c \\ u_{D_1}(i) \leftarrow u_{D_1}(i) + d(i) \\ d(i) \leftarrow d(i) \left[1 - L\phi_1(t_{peak_{D_1}})\right] \end{cases} \tag{14}$$

where $v_{peak_{D_1}}$ is the spike cut-off value, c is the voltage reset value – that is, the value of the membrane potential immediately after the neuron fires. The initial value of $d(i)$, $\forall i = 1, \ldots, N_{D_1}$, is tuned to achieve the desired rate of spiking output. The initial value of $d(i)$ is the same for every neuron i within the population, but as soon as the simulation starts, $d(i)$ may change for each neuron within the population following equation (14). Finally, parameter $L \in [0, 1]$ is a scaling coefficient for the Ca^{2+} current effect.

For the synaptic currents:

$$\begin{aligned} I_{D_1}(t) &= I^*_{pCtx_{NMDA_{D_1}}}(t) - I_{Local}(t) + I_{back_{D_1}}(t), \\ I^*_{pCtx_{NMDA_{D_1}}}(t) &= \left[1 + \beta_1\phi_1(t)\right] I_{pCtx_{NMDA_{D_1}}}(t). \end{aligned} \tag{15}$$

All the currents in (15) are vectors of dimension N_{D_1}. The current $I_{pCtx_{NMDA_{D_1}}}(t)$ models the excitatory contribution of pyramidal neurons with NMDA-receptors of the posterior cortex, and $I_{back_{D_1}}(t)$ is the random background input to the D_1-MSN population. $I_{back_{D_1}}(t)$ is obtained from random values generated with a standard uniform distribution; in this manner, we can induce random neuronal spike trains in our model. $I_{Local}(t)$ models the recurrent activity of MSNs with D_1-type receptors. $\beta_1 \in (0,1]$ is a scaling coefficient of the dopamine's effect.

Each synaptic input current $I_{pCtx_{NMDA_{D_1}}}(t)$, $I_{Local}(t)$ is obtained in an analogous way as in (5)–(8), substituting $S_{NMDA_{pCtx}}$ by S_{D_1}, and considering appropriate dimensions of the connection matrices depending on the number of neurons of the pre- and post-synaptic populations. More details are given in Section 2.

1.2.2 Model for D_2-MSNs

For MSNs with D_2-type receptors, the dynamics for the membrane potential (v_{D_2}) and the recovery current (u_{D_2}) are considered as:

$$\begin{aligned} C\frac{dv_{D_2}(t)}{dt} &= k\big[v_{D_2}(t)-v_r\big]\big[v_{D_2}(t)-v_t\big]-u_{D_2}(t)+I_{D_2}(t)-2\phi_2(t)g_{DA}\big[v_{D_2}(t)-E_{DA}\big], \\ \frac{du_{D_2}(t)}{dt} &= a\Big\{b\big[v_{D_2}(t)-v_r\big]-u_{D_2}(t)\Big\}, \end{aligned} \tag{16}$$

where $v_{D_2} \in \mathbb{R}^{N_{D_2}}$, $u_{D_2} \in \mathbb{R}^{N_{D_2}}$ with N_{D_2} the number of MSNs with D_2-type receptors within the striatum, $v_r \in \mathbb{R}^{N_{D_2}}$ is the resting membrane potential for each neuron of the population and $v_t \in \mathbb{R}^{N_{D_2}}$ is the instantaneous threshold potential for each neuron of the population. The parameter a is the recovery time constant; k and b are derived from the single-neuron frequency-current $(f-I)$ curve by considering the instantaneous firing-rate versus the net synaptic current. Furthermore, $E_{DA} \in \mathbb{R}^{N_{D_2}}$ and $g_{DA} \in \mathbb{R}$ is the reversal potential for each neuron of the D_2-MSN population and the conductance of dopamine regulation, respectively. All the elements of vector E_{DA} are the same.

The function $\phi_2(t)$, with values in $[0,1]$, models the proportion of active dopamine in D_2-type receptors in the MSNs at every time t. It was explained in the previous section and has the form of equation (13).

We consider the following spike-generation and reset conditions of every element i of vectors v_{D_2} and u_{D_2} at $t_{peak_{D_2}}$, when the D_2-MSN fires:

$$\text{for all } i, \text{ if } \; v_{D_2}(i) \geq v_{peak_{D_2}} \; \text{ then} \begin{cases} v_{D_2}(i) \leftarrow c \\ u_{D_2}(i) \leftarrow u_{D_2}(i)+d \end{cases} \tag{17}$$

The input current $I_{D_2}(t)$ is defined as:

$$I_{D_2}(t) = I_{pCtx_{NMDA_{D_2}}}(t) - I^{*}_{Local}(t) + I_{back_{D_2}}(t). \tag{18}$$

All the currents in (18) are vectors of dimension N_{D_2}. The current $I_{pCtx_{NMDA_{D_2}}}(t)$ models the excitatory contribution of pyramidal neurons with NMDA-receptors of the posterior cortex, and $I_{back_{D_2}}(t)$ denotes the random excitatory background input to the striatal D_2-MSNs. $I_{back_{D_2}}$ is obtained from random values generated with a standard uniform distribution. $I^{*}_{Local}(t)$ models the recurrent activity of MSNs with D_2-type receptors.

Each synaptic input current $I_{pCtx_{NMDA_{D_2}}}(t)$, $I^{*}_{Local}(t)$ is obtained with equation (5), substituting $S_{NMDA_{pCtx}}$ by S_{D_2}, and considering appropriate dimensions of the connection matrices depending on the number of neurons of the pre- and post-synaptic populations. In this case, no STDP update is applied and the synaptic strengths are calculated following equation (5) and the steps explained in Section 2.

The values for the parameters for striatal medium spiny neurons used in our model are given in Table 2.

Table 2: Parameters for the models of the MSNs of the striatum. *rand* represents a random value

Parameter	Description	MSN D_1	MSN D_2
N	Number of neurons	200	200
C	Capacitance	50pF	30pF
v_r	Reset potential	-75.9mV	-77mV
v_t	Instantaneous threshold potential	-33.8mV	-44.1mV
a	Recovery time constant	0.04	0.05
k	Tuned for appropriate firing rate	1.13	1.1
b	Tuned for appropriate firing rate	-8	-15
c	Parameter in the reset of the membrane potential	-65	-65
d	Parameter in the reset of the recovery current	700	600
v_{peak}	Spike threshold	40mV	40mV
L	Scaling coefficient of Ca^{2+} current effect	0.831	-
g_{DA}	Conductance of dopamine	21.7	21.1
E_{DA}	Reversal potential of dopamine	-68.4	-88
ϕ_1 (D_1), ϕ_2 (D_2), $\phi_1 = \phi_2 = \phi$	Dopamine receptor occupancy	Variable	Variable
ϕ_{tonic}	Tonic dopamine level	Variable	Variable
ϕ_{phasic}	Phasic dopamine level	Variable	Variable
η	Parameter in expression for dopamine level ϕ	0.7	0.7
β_1	Scaling coefficient of dopamine effect	1	-

1.3 Model of the GPe

The dynamics for neurons of the GPe are described with the same equations as in (1)-(2) substituting $v_{NMDA_{pCtx}}$, $u_{NMDA_{pCtx}}$, $I_{NMDA_{pCtx}}$ and $v_{peak_{NMDA_{pCtx}}}$ by v_{GPe}, u_{GPe}, I_{GPe} and $v_{peak_{GPe}}$, respectively. Now, $v_{GPe} \in \mathbb{R}^{N_{GPe}}$, $u_{GPe} \in \mathbb{R}^{N_{GPe}}$, $I_{GPe} \in \mathbb{R}^{N_{GPe}}$, with N_{GPe} the number of neurons within the GPe. Additionally, we define two different values of k for each GPe neuron i:

$$\begin{cases} \text{if } v_{GPe}(i) \geq v_t \text{ then } k = 6, \\ \text{if } v_{GPe}(i) < v_t \text{ then } b = 0.25. \end{cases} \tag{19}$$

We point out that v_t is the instantaneous threshold potential for GPe neurons. We use different values of v_t for different types of neurons. These values are given in the different tables throughout this document.

The input current I_{GPe} is defined as:

$$I_{GPe}(t) = I_{STN_{GPe}}(t) - I_{D_{2GPe}}(t) - I_{Local}(t) + I_{back_{GPe}}(t). \tag{20}$$

All the currents in (20) are vectors of dimension N_{GPe}. The current $I_{STN_{GPe}}(t)$ models the excitatory contribution of neurons from the STN, $I_{D_{2GPe}}(t)$ is the inhibitory contribution of D_2-type MSNs of the striatum, and $I_{back_{GPe}}(t)$ is the random background activity in the GPe, which is obtained by using a standard uniform distribution. $I_{Local}(t)$ models the recurrent activity of the neurons of the GPe.

Each synaptic input current z (where z is one of STN_{GPe}, D_{2GPe}, *Local*) is obtained with equation (5), substituting $S_{NMDA_{pCtx}}$ by S_{GPe}, and considering appropriate dimensions of the connection matrices depending on the number of neurons of the pre- and post-synaptic populations. In this case, no STDP update is applied and the synaptic strengths are calculated following equation (5) and the steps explained in Section 2.

Typical values for the parameters for GPe neurons are given in Table 3.

1.4 Model of the GPi

The dynamics for neurons of the GPi are described with the same equations as in (1)-(2) substituting $v_{NMDA_{pCtx}}$, $u_{NMDA_{pCtx}}$, $I_{NMDA_{pCtx}}$ and $v_{peak_{NMDA_{pCtx}}}$ by v_{GPi}, u_{GPi}, I_{GPi} and $v_{peak_{GPi}}$, respectively. In this case, $v_{GPi} \in \mathbb{R}^{N_{GPi}}$, $u_{GPi} \in \mathbb{R}^{N_{GPi}}$, $I_{GPi} \in \mathbb{R}^{N_{GPi}}$, with N_{GPi} the number of neurons within the GPi.

Table 3: Parameters for the model of the GPe neurons. *rand* represents a random value

Parameter	Description	GPe
N	Number of neurons	100
C	Capacitance	200pF
v_r	Reset potential	-52mV
v_t	Instantaneous threshold potential	-44.31-$rand$ mV
a	Recovery time constant	1
k	Tuned for appropriate firing rate	0.25
b	Tuned for appropriate firing rate	0.25
c	Parameter in the reset of the membrane potential	-54.22-2$rand$
d	Parameter in the reset of the recovery current	20+5$rand$
v_{peak}	Spike threshold	25mV

Table 4: Parameters for the model of the GPi neurons. *rand* represents a random value

Parameter	Description	GPi
N	Number of neurons	100
C	Capacitance	50pF
v_r	Reset potential	-52+3$rand$ mV
v_t	Instantaneous threshold potential	-44.31+2$rand$ mV
a	Recovery time constant	1
k	Tuned for appropriate firing rate	0.25
b	Tuned for appropriate firing rate	0.25
c	Parameter in the reset of the membrane potential	-54.22+5$rand$
d	Parameter in the reset of the recovery current	20-10$rand$
v_{peak}	Spike threshold	25mV

The input current I_{GPi} is defined as:

$$I_{GPi}(t) = I_{STN_{GPi}}(t) - I_{D_{1GPi}}(t) - I_{GPe_{GPi}}(t) + I_{back_{GPi}}(t). \tag{21}$$

All the currents in (21) are vectors of dimension N_{GPi}. The current $I_{STN_{GPi}}(t)$ models the excitatory contribution of neurons from the STN, $I_{D_{1GPi}}(t)$ is the inhibitory contribution of the D_1-MSNs of the striatum, $I_{GPe_{GPi}}(t)$ is the regulatory and inhibitory input from the GPe and $I_{back_{GPi}}(t)$ is the background activity in the GPi, which is obtained with a Poisson probability distribution.

Each synaptic input current $I_{STN_{GPi}}(t)$, $I_{D_{1GPi}}(t)$, $I_{GPe_{GPi}}(t)$ is obtained with equation (5), substituting $S_{NMDA_{pCtx}}$ by S_{GPi}, and considering appropriate dimensions of the connection matrices depending on the number of neurons of the pre- and post-synaptic populations. In this case, no STDP update is applied and the synaptic strengths are calculated following equation (5) and the steps explained in Section 2.

Typical values for the parameters for GPi neurons are given in Table 4.

1.5 Model of the STN

The dynamics for the excitatory neurons of the STN are described as:

$$\begin{aligned} C\frac{dv_{STN}(t)}{dt} &= k\big[v_{STN}(t) - v_r\big]\big[v_{STN}(t) - v_t\big] - u_{STN}(t) + I_{STN}(t), \\ \frac{du_{STN}(t)}{dt} &= a\Big\{b\big[v_{STN}(t) - v_r\big] - u_{STN}(t)\Big\}, \end{aligned} \tag{22}$$

where $v_{STN} \in \mathbb{R}^{N_{STN}}$, $u_{STN} \in \mathbb{R}^{N_{STN}}$, $I_{STN} \in \mathbb{R}^{N_{STN}}$, with N_{STN} the number of STN neurons. $v_r \in \mathbb{R}^{N_{STN}}$ is the resting membrane potential for each neuron of the population and $v_t \in \mathbb{R}^{N_{STN}}$ is the instantaneous threshold potential for each neuron of the population.

We consider the following spike-generation and reset conditions of every element i of vectors v_{STN} and u_{STN} at $t_{peak_{STN}}$, when the STN neuron fires:

$$\text{for all } i, \text{ if } \; v_{STN}(i) \geq v_{peak_{STN}} \; \text{ then} \begin{cases} v_{STN}(i) \leftarrow c - 0.01 u_{STN}(i) \\ u_{STN}(i) \leftarrow u_{STN}(i) + d \end{cases} \tag{23}$$

To allow STN neurons to represent depolarisation when they are hyperpolarised under a certain potential value, we define two different values for parameter b in equation (22), which will be different for each neuron i of the STN (because b will change depending on the firing conditions of each neuron):

$$\begin{cases} \text{if } v_{STN}(i) \geq v_b \text{ then } b = -5, \\ \text{if } v_{STN}(i) < v_b \text{ then } b = 120, \end{cases} \tag{24}$$

where the constant v_b is the bursting threshold for STN neurons. We also consider two different values for the parameter k in equation (22), which will be different for each neuron i of the STN:

$$\begin{cases} \text{if } v_{STN}(i) \geq v_t \text{ then } k = 12, \\ \text{if } v_{STN}(i) < v_t \text{ then } k = 0.2. \end{cases} \tag{25}$$

The input current $I_{STN}(t)$ is defined as:

$$I_{STN}(t) = I_{pCtx_{NMDA_{STN}}}(t) - I_{GPe_{STN}}(t) + I_{Local}(t) + I_{back_{STN}}(t). \tag{26}$$

All the currents in (26) are vectors of dimension N_{STN}. The current $I_{pCtx_{NMDA_{STN}}}(t)$ models the excitatory contribution of the NMDA neurons of the posterior cortex, $I_{GPe_{STN}}(t)$ is the inhibitory contribution of the neurons from the GPe, $I_{Local}(t)$ models the excitatory recurrent activity of the neurons of the STN. Finally, $I_{back_{STN}}(t)$ is the random background activity in the STN, which is obtained with a Poisson probability distribution.

Each synaptic input current $I_{pCtx_{NMDA_{STN}}}(t)$, $I_{GPe_{STN}}(t)$, $I_{Local}(t)$ is obtained with equation (5), substituting $S_{NMDA_{pCtx}}$ by S_{STN}, and considering appropriate dimensions of the connection matrices depending on the number of neurons of the pre- and post-synaptic populations. In this case, no STDP update is applied and the synaptic strengths are calculated following equation (5) and the steps explained in Section 2.

Typical values for the parameters of STN neurons used in our model are given in Table 5.

Table 5: Parameters of the model for the STN neurons. *rand* represents a random value

Parameter	Description	STN
N	Number of neurons	100
C	Capacitance	200pF
v_r	Reset potential	-52+$rand$
v_t	Instantaneous threshold potential	-44.31-$rand$
a	Recovery time constant	1
k	Tuned for appropriate firing rate	0.25
b	Tuned for appropriate firing rate	0.25
c	Parameter in the reset of the membrane potential	-54.22-2 $rand$
d	Parameter in the reset of the recovery current	20+5 $rand$
v_{peak}	Spike threshold	25mV
v_b	Bursting threshold	-70mV

1.6 Model of the Thalamus

The model of the thalamus only considers excitatory thalamocortical (TC) neurons. The dynamics of these excitatory neurons are described by:

$$
\begin{aligned}
C\frac{dv_{Thl}(t)}{dt} &= k\big[v_{Thl}(t)-v_r\big]\big[v_{Thl}(t)-v_t\big]-\big[u_{Thl_1}(t)+u_{Thl_2}(t)\big]+I_{Thl}(t),\\
\frac{du_{Thl_1}(t)}{dt} &= a_{Ca}\Big\{b_{Ca}\big[v_{Thl}(t)-v_r\big]-u_{Thl_1}(t)+0.75u_{Thl_2}(t)\Big\},\\
\frac{du_{Thl_2}(t)}{dt} &= a_K\Big\{b_K\big[v_{Thl}(t)-v_r\big]-u_{Thl_2}(t)-0.05u_{Thl_1}(t)\Big\},
\end{aligned}
\tag{27}
$$

where $v_{Thl}\in\mathbb{R}^{N_{Thl}}$, $u_{Thl_1}, u_{Thl_2}\in\mathbb{R}^{N_{Thl}}$, $I_{Thl}\in\mathbb{R}^{N_{Thl}}$, with N_{Thl} the number of excitatory thalamic neurons. $v_r\in\mathbb{R}^{N_{Thl}}$ is the resting membrane potential for each neuron of the population and $v_t\in\mathbb{R}^{N_{Thl}}$ is the instantaneous threshold potential for each neuron of the population.

We consider the following spike-generation and reset conditions of every element i of vectors v_{Thl}, u_{Thl_1} and u_{Thl_2} at $t_{peak_{Thl}}$, which is a modification of the original reset conditions from [3]:

$$
\text{for all } i, \text{ if } \ v_{Thl}(i)\ge v_{peak_{Thl}}+0.1u_{Thl_1}(i) \text{ then} \begin{cases} v_{Thl}(i)\leftarrow c_{Thl}-0.1u_{Thl_1}(i)\\ u_{Thl_1}(i)\leftarrow u_{Thl_1}(i)+d_{Ca}\\ u_{Thl_2}(i)\leftarrow u_{Thl_2}(i)+d_K \end{cases}
\tag{28}
$$

Table 6: Parameters for the model for the thalamocortical neurons. *rand* represents a random value

Parameter	Description	Thalamocortical Neurons
N	Number of neurons	100
C	Capacitance	200pF
v_r	Reset potential	-61-2 *rand* mV
v_t	Instantaneous threshold potential	-48-2 *rand* mV
k	Tuned for appropriate firing rate	1.6
a_{Ca}	Recovery time constant	0.05
a_K	Recovery time constant	0.002
b_{Ca}	Tuned for appropriate firing rate	0
b_K	Tuned for appropriate firing rate	0.5
c_{Thl}	Parameter in the reset of the membrane potential	-60-5 *rand*
d_{Ca}	Parameter in the reset of the recovery currents	4
d_K	Parameter in the reset of the recovery currents	2
v_{peak}	Spike threshold	35mV
$v_{b_{Ca}}$	Bursting threshold	-65mV
v_{b_K}	”	-55mV
v_K	”	-20mV

To allow the thalamocortical neurons to have depolarising activity when they are hyperpolarised under a certain potential value, we define two different values for parameters b_{Ca} and b_K in equation (27), which will be different for each thalamocortical neuron i:

$$
\begin{cases} \text{if } v_{Thl}(i)\ge v_{b_{Ca}} \text{ then } b_{Ca}=0,\\ \text{if } v_{Thl}(i)< v_{b_{Ca}} \text{ then } b_{Ca}=45, \end{cases}
\tag{29}
$$

$$
\begin{cases} \text{if } v_{Thl}(i)\ge v_{b_K} \text{ then } b_K=0.5,\\ \text{if } v_{Thl}(i)< v_{b_K} \text{ then } b_K=25, \end{cases}
\tag{30}
$$

where the constants $v_{b_{Ca}}$ and v_{b_K} are bursting thresholds for the thalamocortical neurons. This differentiation is inspired from [3].

Additionally, we consider:

$$\begin{cases} \text{if } v_{Thl}(i) \leq v_K \text{ then } k = 1.6, \\ \text{if } v_{Thl}(i) > v_K \text{ then } k = 25. \end{cases} \tag{31}$$

The input current $I_{Thl}(t)$ is defined as:

$$I_{Thl}(t) = I_{pCtx_{NMDA_{Thl}}}(t) - I_{GPi_{Thl}}(t) + I_{Local}(t) + I_{back_{Thl}}(t). \tag{32}$$

All the currents in (32) are vectors of dimension N_{Thl}. The current $I_{GPi_{Thl}}(t)$ models the inhibitory contribution of neurons from the GPi, $I_{pCtx_{NMDA_{Thl}}}(t)$ represents the cortical excitatory input and $I_{Local}(t)$ models the excitatory recurrent activity of the thalamocortical neurons. Moreover, $I_{back_{Thl}}(t)$ is the random background activity in the thalamus, which is obtained by using a standard uniform distribution.

Each synaptic input current $I_{GPi_{Thl}}(t)$, $I_{pCtx_{NMDA_{Thl}}}(t)$, $I_{Local}(t)$ is obtained with equation (5), substituting $S_{NMDA_{pCtx}}$ by S_{Thl}, and considering appropriate dimensions of the connection matrices depending on the number of neurons of the pre- and post-synaptic populations. In this case, no STDP update is applied and the synaptic strengths are calculated following equation (5) and the steps explained in Section 2.

The values of the parameters for the thalamic neurons considered in our model are given in Table 6.

Finally, and before describing how we connect the different neurons, we provide, in Table 7, the initial values for the membrane potentials and recovery currents used to numerically integrate the differential equations describing the dynamics for all the single neurons.

Table 7: Initial values for the membrane potentials (v_0) and the recovery currents (u_0) at $t = 0$ for each type of neuron in our model. Remark: the values for v_r and b are different for each type of neuron. Their values are provided in Tables 1, 2, 3, 4, 5 and 6

Type of Neuron	Initial v (v_0)	Initial u (u_0)	Initial u_{Thl_1}	Initial u_{Thl_2}
Cortex (NMDA,GABA)	-65mV	bv_0	-	-
Striatum (D_1, D_2)	-86mV	bv_0	-	-
GPe	v_r	bv_0	-	-
GPi	v_r	bv_0	-	-
STN	c	bv_0	-	-
Thalamus	c_{Thl}	-	$b_{Ca}v_0$	$b_K v_0$

2 Modelling the Evolving Interconnections of the Network

In Section 1, we explained the main elements of our basal ganglia network model, especially designed to study Parkinson's disease. The neuron dynamics presented define the evolution of each neuron's behaviour over time and determine the way neurons behave according to inputs. The connections between neurons in the same neural population, and the connections between different populations of neurons represent the evolution of the relationships between them. This evolution depends on the firing activity and firing times of the neurons, and it will be explained in this section.

In Section 1.1.2, we gave details on the evolving connections and the synaptic strength evolution between the NMDA-receptor-type neurons of the posterior cortex. We will use this example to describe how we model all the connections in the proposed network. We will use a generic nomenclature without subscripts to represent the synaptic strenghts of the neurons of our model.

The connections between neurons are defined by means of what we have called a connection matrix S, see equation (5). Let us consider that synapses are connecting neurons of two populations, having pre-synaptic and post-synaptic neurons. For each pre- and post-synaptic population, we define a different connection matrix S. The dimension of the matrix S is $N_{pre} \times N_{post}$, with N_{post} the number of neurons in the post-synaptic population and N_{pre} the number of neurons in the pre-synaptic population. With this, we are establishing that we have an all-to-all topology between the neurons of two populations. However, we will assign a probability to each link between two neurons, and consequently, we will finally build random-graph networks for populations of neurons. Each element (i, j) of the connection matrix S contains the synaptic strength (s) between the i pre-synaptic neuron that has fired at some time t_n and the j post-synaptic neuron. For the pre-synaptic neuron, we also consider the axonal conduction delay t_d, which will be used to update the synaptic strengths. The elements $S(i, j)$, for all $(i, j) \in N_{pre} \times N_{post}$ such that $i = j$, are considered 0 to indicate that it is not possible to have a connection between a neuron and itself.

We do not compute the synaptic strengths for every element of the connection matrices S. The connection probabilities between populations of neurons given in Table 10 determine how sparse the connection matrix S will be. For example, let us come back to Section 1.1.2, and recall the computation of the elements of matrix $S_{NMDA_{pCtx}}$ in order to obtain the recurrent activity of excitatory NMDA-receptor-type neurons in the posterior cortex (I_{local} in (5)). For example, consider that we have 80 NMDA-receptor-type neurons in the posterior cortex. Consequently, the size of $S_{NMDA_{pCtx}}$ is 80×80. Consider that the connection probability between two NMDA-type neurons is 0.2, then there will be 1280 possible connections between these 80 neurons. We will randomly choose these 1280 elements within the matrix $S_{NMDA_{pCtx}}$, and we will calculate their values – that is, the synaptic strengths $s_{NMDA_{pCtx}}$ – in the following manner.

- We will first initialise the value for each synaptic strength s (for example, $s_{NMDA_{pCtx}}$) between a pre- and post- synaptic neuron with the product of the initial synaptic strength, J_s, and a uniformly distributed random number r between 0 and 1. This random scaling parameter r is also used in the computation of some of the synaptic input currents as it is defined in equation (5). The values used for the initial synaptic strength, J_s, are given in Table 9. The random value r provides different initial conditions for each simulation.
- Time-varying STDP-like synaptic plasticity is only considered for the connections within the cortical NMDA-type neurons, the connections between the cortical NMDA-type and GABAergic neurons, and finally, for the connections between the cortical NMDA-type neurons and the D_1-MSNs. For the rest of neurons, we will use expression (5) to compute the synaptic input currents, and the synaptic strengths s will not change over time and will maintain their initial value for all the simulation time.
- For the neurons with time-varying STDP-like synaptic plasticity, once we start simulating our model, the initial values for the synaptic strengths 'evolve' according to equations of the same form as (6), (7) and (8). If a pre-synaptic neuron causes the firing of a post-synaptic neuron, the corresponding synaptic strength s may increase or decrease according to equations (7) and (8). How much we increment or decrease the connection strength is determined by the product of the increment parameter J_{inc} and the parameter ω. $J_{inc} \in (0, 1]$ is a parameter which is the same for each neuron within the same population, and ω is a parameter with a value between 0 and 1 which depends on the dopamine level $\phi(t)$ and is considered as $\omega = \phi(t)/100 + 0.002$, with t the time the pre-synaptic and the post-synaptic neurons fire simultaneously (or they are close to firing together, to be precise). The values used in our simulations for parameter J_{inc} are given in Table 10. Furthermore, if the firing times of the pre- and post-synaptic neurons 'do not coincide' (including t_d for the pre-synaptic neuron), the synaptic strength stays the same according to equation (6).

The synaptic strengths can have positive or negative values. However, we will limit the maximum and minimum values of these synaptic strengths for the ones varying over time following the STDP model, such that $|s| \leq s_{max}$. In this manner, we avoid instabilities in the simulation. Values for s_{max} are given in Table 10 for each time-varying synaptic connection.

In this context, an existing connection between the corresponding pre-synaptic and post-synaptic neuron may disappear (the value of the synaptic strength may become 0, $s = 0$) or a non-existent connection may be formed (the value of the synaptic strength is different from 0) depending on the spike times. This is what we call *evolution* of the connections. With this,

Table 8: Interconnections between the neural populations

Connection (Pre-synaptic)	Number of neurons	Targets (Post-synaptic)
$PosteriorCortexNMDA$	800	Local, Posterior Cortex (GABA), $MSNs_{D1}$, $MSNs_{D2}$, STN, Thalamic Cells
$PosteriorCortexGABA$	200	Posterior Cortex (NMDA)
$MSNs_{D1}$	200	Local, GPi
$MSNs_{D2}$	200	Local, $MSNs_{D1}$, GPe
GPi	100	Thalamic Cells
GPe	100	Local, GPi, STN
STN	100	Local, GPi, GPe
$ThalamicCells$	100	Local

Table 9: Parameters for the synaptic connections between neurons. The initial synaptic strength (J_s) will not change over time for connections without STDP

Connection (From-To)	Number of synapses per neuron	Initial synaptic strength (J_s)	Axonal conduction delay (t_d)
$pCtx_{NMDA} \to pCtx_{NMDA}$	100	100	20ms
$pCtx_{NMDA} \to pCtx_{gaba}$	100	100	20ms
$pCtx_{NMDA} \to MSN_{D1}$	80	175	11ms
$pCtx_{NMDA} \to MSN_{D2}$	50	150	15ms
$pCtx_{NMDA} \to STN$	35	100	6ms
$pCtx_{NMDA} \to TC$	40	75	25ms
$pCtx_{gaba} \to pCtx_{NMDA}$	100	-50	20ms
$MSN_{D1} \to MSN_{D1}$	30	-10	5ms
$MSN_{D1} \to GPi$	60	1200	6ms
$MSN_{D2} \to MSN_{D1}$	30	-5	5ms
$MSN_{D2} \to MSN_{D2}$	30	-5	5ms
$MSN_{D2} \to GPe$	50	1100	6ms
$GPi \to TC$	75	150 (for PD-related case 3.5)	2ms
$GPi \to TC$	75	600 (for 3.1, 3.2, 3.3, 3.4 cases)	2ms
$GPe \to GPe$	20	45	2ms
$GPe \to STN$	40	125	6ms
$GPe \to GPi$	20	100	6ms
$STN \to STN$	20	50	3ms
$STN \to GPi$	40	400	2ms
$STN \to GPe$	45	100	2ms
$TC \to TC$	30	200	5ms

Table 10: Parameters for the synaptic connections between neurons

Connection (From-To)	Max. synaptic strength (s_{max})	Increment of the synaptic strength (J_{inc})	Connection probability %
$pCtx_{NMDA} \to pCtx_{NMDA}$	250	0.03	10
$pCtx_{NMDA} \to pCtx_{gaba}$	250	0.03	12.5
$pCtx_{NMDA} \to MSN_{D1}$	400	0.1	40
$pCtx_{NMDA} \to MSN_{D2}$	-	-	25
$pCtx_{NMDA} \to STN$	-	-	40
$pCtx_{NMDA} \to TC$	-	-	40
$pCtx_{gaba} \to pCtx_{NMDA}$	250	0.03	10
$MSN_{D1} \to MSN_{D1}$	-	-	15
$MSN_{D1} \to GPi$	-	-	60
$MSN_{D2} \to MSN_{D1}$	-	-	7.5
$MSN_{D2} \to MSN_{D2}$	-	-	7.5
$MSN_{D2} \to GPe$	-	-	50
$GPi \to TC$	-	-	75
$GPe \to GPe$	-	-	20
$GPe \to STN$	-	-	40
$GPe \to GPi$	-	-	20
$STN \to STN$	-	-	20
$STN \to GPi$	-	-	40
$STN \to GPe$	-	-	45
$TC \to TC$	-	-	30

our model can capture what is known as *structural plasticity*. That is, not only is synaptic behaviour modified, but synapses may also be rewired. This concept of structural plasticity can be also associated to changes in the network topology, which is also termed *wiring plasticity*.

For the simulations, we consider the following set up. The cortical network consists of two different types of sub-populations: excitatory and inhibitory. We use the ratio of excitatory-to-inhibitory number of neurons of 4:1 (800:200 neurons) for the posterior cortex. In the striatum, we consider 200 striatal D_1-MSNs and 200 D_2-MSNs. For the rest of the neural populations, we consider 100 neurons. As we mentioned above, all the neurons in the model are connected according to a random graph, and the initial probabilities in the local connections within a population, and the interconnections between neurons of different populations, are chosen in

accordance to neurophysiological studies reported in the literature. Some of the connection probabilities are chosen to be higher to allow the pre-synaptic population to drive the post-synaptic population more accurately. We also make some of the inhibitory synaptic couplings stronger than some of the excitatory ones to allow the inhibitory neurons to adequately regulate the excitatory populations behaviour. The number of neurons in each pre-synaptic population and the list of the connections are given in Table 8.

References

1. Çelikok U, Navarro-López EM, Şengör NS (2016) A computational model describing the interplay of basal ganglia and subcortical background oscillations during working memory processes. ArXiv:1601.07740 [q-bio.NC], http://arxiv.org/abs/1601.07740.
2. Humphries M, Lepora N, Wood R, Gurney K (2009) Capturing dopaminergic modulation and bimodal membrane behaviour of striatal medium spiny neurons in accurate, reduced models. *Frontiers in Computational Neuroscience* 3(26):1-16.
3. Izhikevich EM (2007) Dynamical systems in neuroscience: the geometry of excitability and bursting. MIT Press, Cambridge, MA.
4. Navarro-López EM, Çelikok U, Şengör NS (2016) Hybrid systems neuroscience. In: El Hady A (ed) Closed-loop neuroscience. Academic Press, pp 113-129.
